# Green nanotechnology for controlling bacterial load and heavy metal accumulation in Nile tilapia fish using biological selenium nanoparticles biosynthesized by *Bacillus subtilis* AS12

**DOI:** 10.3389/fmicb.2022.1015613

**Published:** 2022-12-23

**Authors:** Ahmed M. Saad, Mahmoud Z. Sitohy, Mohamad I. Sultan-Alolama, Khaled A. El-Tarabily, Mohamed T. El-Saadony

**Affiliations:** ^1^Biochemistry Department, Faculty of Agriculture, Zagazig University, Zagazig, Egypt; ^2^Department of Biology, College of Science, United Arab Emirates University, Al Ain, United Arab Emirates; ^3^Department of Health, Research and Innovation Center, Zayed Complex for Herbal Research and Traditional Medicine, Abu Dhabi, United Arab Emirates; ^4^Khalifa Center for Genetic Engineering and Biotechnology, United Arab Emirates University, Al Ain, United Arab Emirates; ^5^Harry Butler Institute, Murdoch University, Murdoch, WA, Australia; ^6^Department of Agricultural Microbiology, Faculty of Agriculture, Zagazig University, Zagazig, Egypt

**Keywords:** *Bacillus*, bacterial load, behavior, Bio-SeNPs, fish, growth, heavy metals, performance

## Abstract

Heavy metal accumulation and pathogenic bacteria cause adverse effects on aquaculture. The active surface of selenium (Se) nanoparticles can mitigate these effects. The present study used Se-resistant *Bacillus subtilis* AS12 to fabricate biological Se nanoparticles (Bio-SeNPs). The double-edged Bio-SeNPs were tested for their ability to reduce the harmful effects of heavy metals and bacterial load in Nile tilapia (*Oreochromis niloticus*) and their respective influences on fish growth, behavior, and health. The Bio-SeNPs have a spherical shape with an average size of 77 nm and high flavonoids and phenolic content (0.7 and 1.9 g g^−1^ quercetin and gallic acid equivalents, respectively), resulting in considerable antioxidant and antibacterial activity. The Bio-SeNPs (3–5 μg ml^−1^) in the current study resolved two serious issues facing the aquaculture industry, firstly, the population of pathogenic bacteria, especially *Aeromonas hydrophilia*, which was reduced by 28–45% in fish organs. Secondly, heavy metals (Cd and Hg) at two levels (1 and 2 μg ml^−1^) were reduced by 50–87% and 57–73% in response to Bio-SeNPs (3–5 μg ml^−1^). Thus, liver function parameters were reduced, and inner immunity was enhanced. The application of Bio-SeNPs (3–5 μg ml^−1^) improved fish gut health, growth, and behavior, resulting in fish higher weight gain by 36–52% and a 40% specific growth rate, compared to controls. Furthermore, feeding and arousal times increased by 20–22% and 28–53%, respectively, while aggression time decreased by 78% compared to the control by the same treatment. In conclusion, Bio-SeNPs can mitigate the accumulation of heavy metals and reduce the bacterial load in a concentration-dependent manner, either in the fish media or fish organs.

## Introduction

Fish and fish products are important sources of amino acids and micronutrients for health and a well-balanced diet. Rising demands for animal protein are usually met with an increase in intensive farming and production, which contributes to negative environmental effects. However, aquaculture faces increased challenges due to exotic and endemic epizootic diseases and the shifting climate (Abu-Elala et al., 2020).

Many fish farms use agricultural drainage water, which is usually full of fertilizers, pesticides, and toxic heavy metals such as cadmium (Cd), mercuric (Hg), lead (Pb), copper (Cu), and zinc (Zn; Omar et al., 2012). Bosch et al. (2016) found that fish products usually have several heavy elements, especially Hg and other pollutants. For example, Cd is both non-biodegradable and non-digestible and is consequently directly integrated into the food chain (Bosch et al., 2016). Hg tightly binds to proteins within fish tissues, where it hyper concentrates, often as “methylmercury,” a highly toxic organic compound. Cd and Hg induce free radicals in fish, damaging antioxidative defenses and increasing lipid peroxidation (Negahdari et al., 2021).

Also, Cd and Hg are toxic to the immune and nervous systems, genes, kidneys, and cells and cause cancer in other organisms, including humans (El Nemr et al., 2016). There are ongoing studies in search of novel antioxidants and antimicrobial agents with the potential to provide fish with the required nutrients to simultaneously improve growth performance while also promoting their defense and immunological systems (Dawood et al., 2019).

Selenium (Se) is a fundamental element in enzymatic and non-enzymatic systems that protects the cellular membrane against oxidative damage (Talas et al., 2008). Additionally, it is a vital micronutrient in fish food, promoting growth and fertility (Longbaf Dezfouli et al., 2019). Despite Se′s physiological, biochemical, and immunological benefits for fish, it is a highly toxic element (Mechlaoui et al., 2019). Nanotechnology offers novel solutions for making selenium safe and bioavailable for aquatic organisms in the form of selenium nanoparticles (SeNPs) because of the small size of Se in nanoparticles (Shaalan et al., 2020; El-Adawy et al., 2021; Lang et al., 2021).

SeNPs have shown outstanding bioavailability and low toxicity levels (Menon et al., 2018). They can exert envisioned activity at much lower concentrations than organic or inorganic Se compounds. Further, SeNPs exhibit biological activities that promote optimum substitutions for various seleno-compounds (Yanhua et al., 2016). Including SeNPs in fish diets releases safe Se, which acts as a nutritional additive and promotes growth performance (Ren et al., 2021).

There are many routes to produce SeNPs, including chemical and physical methods. SeNPs have a low cytotoxicity level, as the nanoparticles destroy cancerous cells by releasing more reactive oxygen species (ROS) into cancer cells rather than normal cells. Therefore, it is being investigated for integration into nanomedicine applications for accelerating wound healing (Kumar et al., 2015; Skalickova et al., 2017).

However, these methods have many problems, i.e., toxic chemicals and high energy consumption. Therefore, nanoparticles have emerged as eco-friendly and green synthesis methods (Salem, 2022a). High pressure or temperature is not required for the green synthesis of NPs, and the use of toxic and hazardous substances and the addition of external reducing, stabilizing, or capping agents are avoided (Salem and Fouda, 2021).

Several studies used bacteria for biological synthesis of nanoparticles, i.e., *Lactobacillus paracasei* HM1 (El-Saadony et al., 2021a), *Bacillus subtilis* AL43 (Abdel-Moneim et al., 2022), and *Lactobacillus acidophilus* ML14 (El-Saadony et al., 2021b). Additionally, fungi such as *Aspergillus oryzae* (Mosallam et al., 2018) and *Mariannaea* sp. were also used (Zhang et al., 2019).

This “green” (biological) synthesis of SeNPs ensures its safe use in many biomedical applications, with no side effects (Alagesan and Venugopal, 2019). Also, it was used in water and wastewater treatment, carbon nanotubes, metal oxides (Salem et al., 2022a), and in agriculture as nano-fertilizers (El-Saadony et al., 2021e). SeNPs exhibited antioxidant, antimicrobial, antiviral, and anti-tumor activities (Hariharan and Dharmaraj, 2020). They also have insecticidal activity (Salem et al., 2021; Hashem et al., 2022) and have antifouling against *Pseudomonas aeruginosa*, *Staphylococcus aureus*, and *Candida albicans* (Abu-Elghait et al., 2021; Salem et al., 2022b).

SeNPs are protective agents against heavy metals such as Chromium (Cr), Cd, and Hg, as well as chemotherapeutic agents with adverse effects through the generation of ROS. SeNPs may activate caspase-3 and enhance poly (ADP-ribose) polymerase-1 (PARP) cleavage, leading to mitochondria-mediated apoptosis through stabilizing DNA and neutralizing ROS production (Kiełczykowska et al., 2018; Khurana et al., 2019; Sonkusre, 2020).

The SeNPs, produced from Na_2_SeO_3_ or seleno-methionine, have been reported to promote efficient growth performance and antioxidant defense systems of *Cyprinus carpio* based on their bioavailability (Saffari et al., 2018). SeNPs enhance physiological parameters (lysozyme activity, hemoglobin concentration, red blood cells (RBC) counts, and hematocrit value). Besides, promoting biochemical parameters in fish organs growth hormone level in serum, total proteins in tissue, and peroxidase and glutathione activities in the liver and muscles (Khan et al., 2016).

Dawood et al. (2019) confirmed that SeNPs promoted the immune system in Red Sea Bream by increasing skin mucus, serum protein, and reducing salinity tolerance by increasing the populations and activities of T-cells and NK cells (Avery and Hoffmann, 2018). Simultaneously, Se was reported to have substantial antioxidant activity (Saffari et al., 2017).

No studies yet, have investigated the effects of double-edged Bio-SeNPs synthesized by bacteria to simultaneously reduce the harmful effects of heavy metals and microbial load on Nile tilapia (*Oreochromis niloticus*) and to delineate their influences on fish growth, behavior, and health. In continuation with the authors’ previous research on green nanotechnologies (El-Saadony et al., 2021c), the current study attempts to introduce new green nanotechnology; in addition, the optimum conditions for producing Bio-SeNPs and the transformation of Bio-SeNPs was chemically and biologically evidenced, then characterized. Furthermore, we demonstrated their antioxidant and antibacterial properties. Consequently, Bio-SeNPs were included in Nile tilapia media to counteract heavy metal and microbial accumulation in fish. Then light was casted on fish growth and immunological effects of dietary Bio-SeNPs. The prospective role of Bio-SeNPs in mitigating oxidative status and the adverse effects of toxic Hg and Cd, and the microbial contamination on Nile tilapia was also studied.

## Materials and methods

### Soil samples, bacteriological media, and bacterial type strains

Soil samples were collected in sterile bags from soils polluted with industrial wastewater near the Ismailia Canal (30°0.11′02.054” N, 31°0.27′87.466″ E), Ismailia, Egypt.

All bacteriological media, Plate Count Agar (PCA), nutrient broth (NB), Luria-Bertani (LB), enrichment medium (EM), plate count broth (PCB), and tryptic soy broth (TSB), Mueller Hinton agar (MHA), Mueller Hinton broth (MHB) were purchased from Lab M Limited, Lancashire, United Kingdom. All other chemicals; NaCl, sodium selenite (Na_2_SeO_3_), HCl, potassium iodine, starch indicator 1,1-diphenyl-2-picrylhydrazyl (DPPH), ethanol, 2,2-azino-bis-3-ethylbenzothiazoline-6-sulfonic acid (ABTS), peptone, and Tret-Butyl hydroquinone (TBHQ) were of analytical grade and obtained from Sigma-Aldrich Chemie GmbH, Taufkirchen, Germany.

The bacterial isolates, *Staphylococcus aureus*, *Bacillus cereus*, *Listeria monocytogenes*, *Escherichia coli*, *Aeromonas hydrophilia*, and *Klebsiella pneumonia* were obtained from the Agricultural Microbiology Department, Faculty of Agriculture, Zagazig University, Zagazig, Egypt.

Luria-Bertani (LB) broth [prepared by dissolving 10 g tryptone, 5 g yeast extract, and 5 g sodium chloride in 1000 ml distilled water (pH 7.5)]. Enrichment medium (EM) broth (was prepared by dissolving 0.5 g sodium nitrate, 5 g sodium chloride, 0.1 g ammonium chloride, 2.7 g di-potassium hydrogen phosphate, 3 g tryptone, 1 g beef extract, 0.5 g yeast extract, and 3 g glucose in 1000 ml distilled water).

### Isolation, screening, and identification of Se-resistant bacteria

For selecting Se-tolerant bacterial isolates, the collected soil samples were stored at 4°C until transport to the laboratories of the Agricultural Microbiology Department, Faculty of Agriculture, Zagazig University, Egypt, for immediate processing. The soil samples were used to isolate selenium-resistant bacteria. Twenty-five grams of soil samples were homogenized in of sterile saline peptone water (1 g l^−1^ peptone and 8.5 g l^−1^ NaCl) for 10 min at 25°C to prepare a 10−1 dilution.

One ml of the previous dilution was added to a 9 ml sterile saline peptone water tube to obtain a 10^−2^ dilution. Further serial dilution to 10^−7^. Aliquots (100 μl) of each dilution was spread across the surface of Plate Count Agar (PCA) plates supplemented with 1, 2, 3, 4, 5, and 6 mM Na_2_SeO_3_ in sterilized plastic petri dishes (90 mm diameter) using sterilized L-shaped spreaders. For each dilution, three plates were used for each sample. The plates were incubated for 1 day at 30°C; the red colonies were then selected based on their efficiency in reducing sodium selenite (Srivastava and Kowshik, 2016).

Selenium-tolerant bacteria were identified by microscopic examination. The microscopic images of bacteria were compared with the morphological and biochemical tests defined in the Bergy manual. Further identification was made by MALDI-TOF spectroscopy (Bruker Daltonics, Bremen, Germany). Phylogenetic analysis was performed using the neighbor-joining method (Saitou and Nei, 1987). The MEGA-X software was used to reconstruct a neighbor-joining tree (Tamura et al., 2004; Kumar et al., 2018) using data imported from the GenBank database.^1^

### Biosynthesis and optimization of Bio-SeNPs

An aliquot (100 μl) of the selected bacterial suspension (10^8^ CFU) was inoculated in 100 ml of Luria-Bertani (LB) broth and incubated in a shaking incubator at 30°C and 180 rpm for 48 h. It was then centrifuged at 8,000 rpm for 20 min. The supernatant was discarded, and the bacterial pellets were homogenized in 100 ml of the Enrichment medium (EM) broth supplemented with 5.0 mM of Na_2_SeO_3_. The mixture was then incubated at 30°C for 3 days in a shaking incubator of 180 rpm. The conversion in the flask color (EM medium containing Na_2_SeO_3_ and the tested bacterium) from bright yellow to red at the end of the incubation period confirms the transformation of Na_2_SeO_3_ to Bio-SeNPs by the selected isolate. The flask that contains only Na_2_SeO_3_ and the flask that contains only bacterial pellets without Na_2_SeO_3_ (5 mM) were kept under the same conditions. The Bio-SeNPs were extracted from the bacterial pellets through autoclaving until the explosion of the bacterial membrane; the resulting solution containing Bio-SeNPs was centrifuged at 15,000 rpm for 30 min, the supernatant was subsequently obtained, and the exploded cells were suspended (Fesharaki et al., 2010).

The biosynthesis of Bio-SeNPs were optimized individually using several parameters, namely: (i) various mediums, including NB, LB, EM, PCB, and TSB; (ii) reaction time on different days (1, 2, 3, 4, and 5 days); (iii) temperature (15, 20, 25, 30, and 35°C); (iv) pH (5, 6, 7, 8, and 9); and (v) and agitation speed (100, 140, 180, 220, and 260 rpm). The particle size of the Bio-SeNPs was estimated using the dynamic light scattering (DLS) technique.

### Chemical status of selenium in Bio-SeNPs

A specific amount (1.9 g) of Na_2_SeO_3_ was homogenized in 1,000 ml distilled water to prepare a standard stock solution. The HCl (2 N) solutions and potassium iodine 2% were prepared as a starch indicator. Additionally, numerous concentrations of stock solutions were prepared. An aliquot (100 μl) of stock solution was placed in test tubes before adding 1 ml of HCl and 1 ml of potassium iodine 2% and homogenizing until a brown-yellow color appeared. The Se content was calculated by applying the absorbance in a standard curve equation y = 0.6633x + 0.0015 to acquire the amount in Bio-SeNPs. The same conditions were applied, in parallel, to Na_2_SeO_3_ and Bio-SeNPs. The starch indicator was added to all tubes. The obtained color was read at 644 nm using a microtiter plate reader (BioTek Elx808, United States).

### Physico-chemical characterization of Bio-SeNPs

Bio-SeNPs were characterized according to Abdel-Moneim et al. (2022) and El-Saadony et al. (2021a). The shape and average size of Bio-SeNPs were assessed by transmission electron microscopy images (TEM; JEOL 1010, Japan). The absorption spectra of the synthesized Bio-SeNPs were carried out by UV–visible spectrophotometer in the range of 200–1,000 nm (Mettler-Toledo LLC., Columbus, OH, USA). The exact size and surface charge of Bio-SeNPs were estimated using a Zeta sizer analyzer and the Zeta potential, respectively (Nano Z2 Malven, Malvern Hills, United Kingdom). The active components in Bio-SeNPs were detected by Fourier transform-infrared (FTIR) spectroscopy (Bruker Tensor 37, Kaller Germany) at a wavelength range of 500–3,500 cm^−1^.

### Biological activity of Bio-SeNPs

#### Antioxidant activity

##### DPPH assay

The radical scavenging activity of Bio-SeNPs at different concentrations (0.5, 1, 2, 3, 4, and 5 μg ml^−1^) was estimated according to Bhakya et al. (2016), with some modificationss. A volume (100 μl) of each concentration was mixed with an equal volume of DPPH in ethanol and placed in the microtiter plate. Absorbance was read after 30 min, at 517 nm, using a microtiter plate reader (BioTek Elx808, United States). The absorbance was then applied using Eq. (1) as follows:


(1) $$\begin{array}{l} Radicalscavengingactivity\;\left(\%\right)\\ =\frac{\left(controlabsrobance-sampleabsorbance\right)}{controlabsrobance}\times 100 \end{array}$$

##### ABTS assay

The antioxidant activity of Bio-SeNPs was estimated following the methods outlined by Gil et al. (2002), with slight modifications. Briefly, 100 μl of Bio-SeNPs concentrations (0.5, 1, 2, 3, 4, and 5 μg ml^−1^) were homogenized in an equal volume of 0.1 mM ABTS, in a microtiter plate. The absorbance was subsequently estimated after 30 min at 745 nm, using a microtiter plate reader (BioTek Elx808, United States).

TBHQ and ABTS were used as controls. The % inhibition of radicals was calculated using Eq. (2).


(2) $$\%RSA\;Inhibition=\frac{A\;control-A\;sample}{A\;control}\times 100$$

#### Antibacterial activity

##### Disc assay method

The antibacterial activity of Bio-SeNPs was estimated using the disc diffusion method (Saad et al., 2021). In brief, 100 μl of bacterial inoculum (1×10^8^ CFU ml^−1^) was spread over the surface of an MHA plate. Sterilized paper discs (6 mm) were prepared and saturated with Bio-SeNPs concentrations of (0.5, 1, 2, 3, 4, and 5 μg ml^−1^), then placed at the sides of MHA plates. Discs saturated with sterilized distilled water served as the control. All plates were incubated at 37°C for 1 day. The inhibition zones around the discs were measured and recorded (mm).

##### Minimum inhibitory concentration and minimum bactericidal concentration

The control was a tube containing 9 ml of Mueller Hinton Broth (MHB) inoculated with the bacterial inoculum. A 500 μl of each Bio-SeNPs level was mixed in 9 ml of MHB tubes previously inoculated with 500 μl of the bacterial inoculum. All tubes were incubated for 24 h at 37°C. The minimum inhibitory concentration (MIC) and the minimum bactericidal concentration (MBC) of Bio-SeNPs were estimated following Saad et al. (2021). The MIC was recorded as the lowest concentration of Bio-SeNPs, inhabiting bacterial growth. The MBC of Bio-SeNPs was determined by spreading 100 μl of MIC tubes over new MHA plates while observing the bacterial growth after a day of incubation at 37°C.

### Fish experimental design

#### Ethical approval

This trial was carried out at the Fish Research Unit, Faculty of Veterinary, Cairo University, Egypt. The total duration of this trial was 10 weeks. All procedures in the study followed international ethical standards. The research involved no human participants. All experimental groups were fed on a basal diet.

#### Experimental material and conditions

A group of 120 healthy (The fish was judged healthy from its overall normal morphology, feeding behavior, active breathing, active movement, brightness, and clear eyes) Nile tilapia (*Oreochromis niloticus*) fish with an initial weight (60 g) were purchased from the Fish Research Central Lab., Ministry of Agriculture, Egypt. Fish were acclimated in glass aquaria containing dechlorinated water for 2 weeks. Water parameters were checked weekly. The quality of water General water parameters were monitored and stabilized; the levels of sodium, potassium, magnesium, and calcium in the water were adjusted to 0.4, 0.05, 0.6, and 0.8 mmol L-1, respectively. At a temperature of 25.4 ± 0.3°C, a dissolved oxygen level of 7.30 ± 0.13 mg/l, a pH of 7.90 ± 0.06, a total hardness level of 132 ± 4.4 mg CaCO3/L, ammonia (NH3) from 0.03 to 0.038 mg/l, total salinity of 23.41 ppt, total ammonia nitrogen 0.004 ± 0.001 g/l, and a photoperiod regime (12:12 h light: dark).

During acclimatization and experimentation, all groups were fed on a basal diet (20% soybean meal, corn 23.0%, fish meal 15.0%, alfalfa hay 14.0%, wheat bran 13.0%, corn gluten meal 11.5%, sunflower oil 1, 1% vitamin mixture, 0.5% mineral mixture, and 1% carboxymethyl cellulose). The approximate analysis of the diet was 30.32% crude protein, 3.53% crude fibers, 1.79% lysine, 0.74% methionine, 0.97% calcium, and 0.86% available phosphorus, with a 9.91% energy expenditure, and a total of 2,905.93 Kcal/Kg of digestible energy.

**SCHEME 1:**
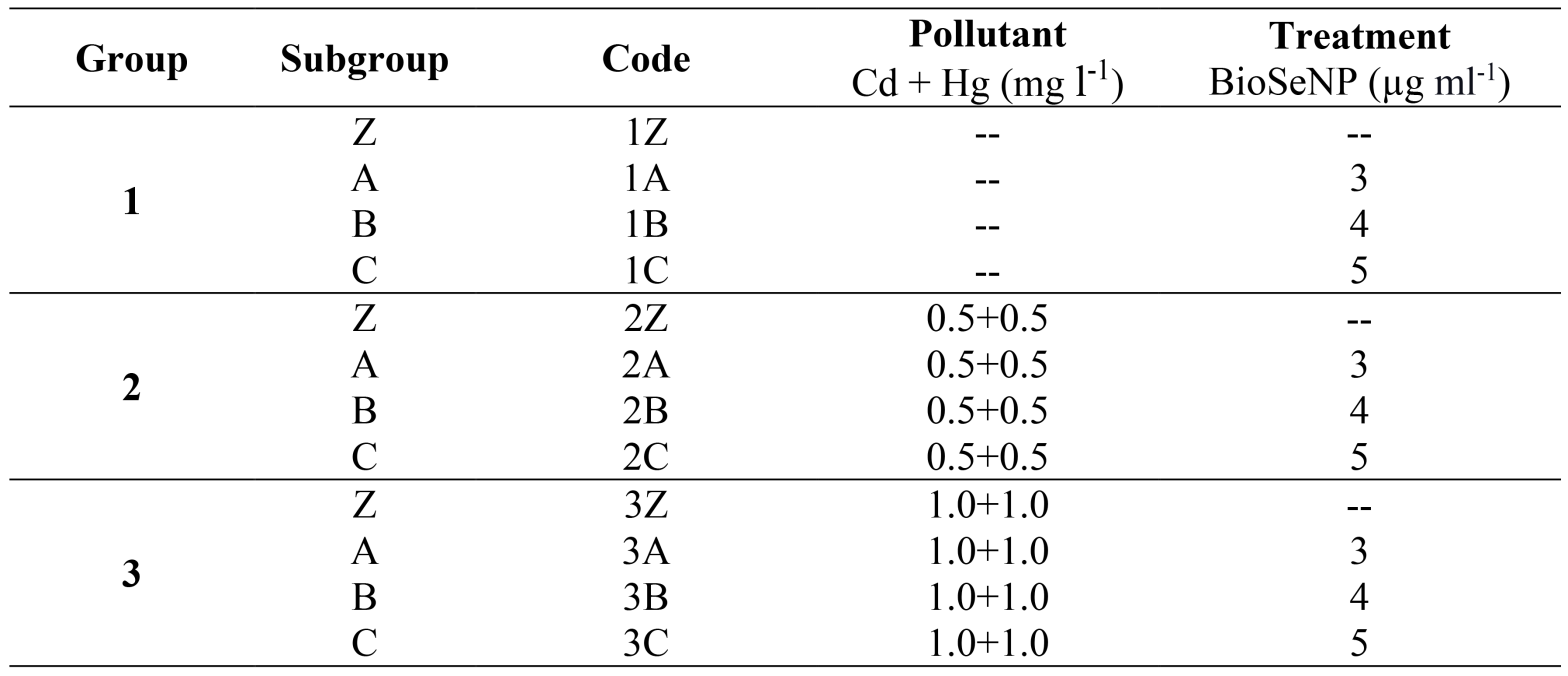
Experimental design.

A group of 120 healthy Nile tilapia fish with an initial weight of 60 g was divided into three main groups; the first group was kept in freshwater, while the second and third groups were subjected to heavy metal-contaminated water (Cd and Hg), respectively. Each group was further subdivided into four subgroups (Z, A, B, and C), each receiving Bio-SNP at 0, 3, 4, and 5 μg ml^−1^. The subgroups for group 1 were 1Z, 1A, 1B, and 1C (Scheme 1). This nomination was consistent for all three groups, with the first digit referring to the group and the second letter to the subgroup. The control in the first group (1Z) was incubated in tap water and received neither heavy metals nor Bio-SeNPs. In contrast, the controls of the second and third groups (2Z and 3Z) received heavy metals at 0.5 + 0.5 Hg mg/L and 1.0 + 1.0 Cd + Hg mg/L, respectively, but without any treatment (Scheme 1). Experimental fish were treated as previously described for the experimental period, a total of 10 weeks (Section “Antioxidant activity”).

The doses of Cd and Hg were inferior to the maximum permissible limit approved by the FDA. The fish were exposed to a semi-static water system through a daily siphoning of 60% in the morning and 40% in the evening, then replaced with fresh water the following day. Levels of the heavy metals and Bio-SeNPs were re-adjusted to the starting values.

#### Fish behavior

Fish were marked with short plastic strips applied to the dorsal fin. The observation technique followed a focal sample procedure for 15 s intervals during 1 h videotaping using a software observer (Phillips et al., 2018). The observed behavior pattern was recorded for all tested behavioral patterns 3 h weekly. Additionally, the mean time and frequency of each behavior were recorded.

Fish behavior was estimated according to the methods described by Neetha et al. (2022). Feeding behavior was recorded based on methods described by Alonso and Valle-Torres (2018). Foraging behavior was defined by fish searching for food while “grazing” within the aquarium. Surfacing behavior was distinguished by how often and how long the fish rose to the surface to breathe. The swimming behavior of fish included rapid and slow movement in the water without any other activities (Bégout Anras et al., 2004). The resting behavior was estimated when fish were not active in the aquaria and without an open eye (Sekiguchi and Kohshima, 2003). Body care behavior includes the time and frequency of lateral body shaking as either rapid movements or scratching of the body against any object (Coppedge et al., 1997; Sekiguchi and Kohshima, 2003).

Aggressive behavior was defined as fish vigorously chasing another group of fish (Molano et al., 2013). Agonistic behavior was considered fin tugging, i.e., biting another fish’s fin and butting the genital papilla. In addition, arousal behavior included movement activity without actual swimming or surfacing behavior.

#### Fish performance

Five fish per aquarium were collected, washed, and weighed for the final body composition at 20°C. Performance traits, including weight gain (WG), percentage specific growth rate (SGR) per day, total feed intake (TFI), feed conversion ratio (FCR), and percentage survival rate (SR), were calculated according to Eqs. 3–7.


(3) WG(g)=W2−W1

(4) $$SGR\;\left(\%day\right)=\frac{100\left[Ln\;W2-LnW1\right]}{T}\;$$

(5) $$\begin{array}{l} TFI\left(g\frac{feed}{fish}\right)\\ =\frac{Thetotalamountsoffishdiets\;}{fishnumberthroughouttheexperimentalperiod}\; \end{array}$$

(6) $$FCR=\frac{TFI}{WG}\;$$

(7) $$\begin{array}{l} SR\;\left(\%\right)\\ =\frac{Fishnumbers\;at\;the\;end\;oftheexperiment}{Fishnumber\;at\;thebeginningoftheexperiment}\;\times 100\; \end{array}$$

Initial weight (W1) and final weight (W2), as well as acclimation days (T) were recorded during the feeding duration (70 days).

#### Fish and water sampling

Three fish per aquarium were dissected, and their skin, muscle, intestine, and gills were removed and kept at −20°C for microbial analysis. Another three fish per aquarium were collected for detecting Cd and Hg content, then the liver, intestine, gills, and kidney were extracted and kept in formalin (10%) until reaching the laboratory. The water samples were collected daily from the aquarium, then homogenized to take a representative sample for analysis.

#### Physiological and biochemical parameters

For the biochemical study, three fish per aquarium were collected. Then, 3 ml of caudal vein blood samples were taken using a heparinized needle and kept in sterile Eppendorf tubes at 4°C. All tubes were centrifuged (Sigma 3-30 K, Germany) at 10,000 rpm for 5 min at 4°C to collect sera. All blood biochemical parameters were measured using kits from Bio-Diagnostic Co. (Dokki, Cairo, Egypt) as described by Al-Deriny et al. (2020) and Dawood et al. (2020). All parameters were estimated at a respected wavelength using a microtiter plate reader (BioTek Elx808, United States).

A nitro blue tetrazolium (NBT) reagent was used to determine respiratory system failure. The levels of blood glucose (GLU), and liver biomarkers, i.e., alanine aminotransferase (ALT), and aspartate aminotransferase (AST), were measured. Additionally, the activities of lysozyme (LYZ), testosterone, progesterone, follicle-stimulating hormone (FSH), growth hormone (GH), and cortisol were evaluated in sera.

#### Heavy metal content in fish organs

Frozen fish samples were thawed and dissected with stainless steel instruments to separate fish organs, liver, kidneys, gills, and muscles. A 5 g of fish organs were taken and digested with concentrated nitric acid for all organs except the gills with a nitric and perchloric acid (4:1) mixture at a temperature of 100°C until reaching a transparent solution.

Cd and Hg were estimated using an atomic absorption Spectrophotometer (Model AAS GPC A932, Version 1.1). The obtained solution was diluted to a known volume with deionized distilled water. Analytical results of fish organs indicated a suitable performance of heavy metal determination within the range of certified values, with a 95–111% recovery for the metals studied (Murtala et al., 2012).

#### Total bacterial and *Aeromonas* spp. count in water and fish tissues

Whole fish (i.e., body including skins, gills, intestines, and muscles) were collected with water samples from aquaria at weekly intervals at 1, 4, 8, and 10 weeks to determine the total bacterial count and *Aeromonas* count. A total of 10 g of fish samples were homogenized in 90 ml of sterilized peptone buffer. This solution was decimally diluted up to 10^−7^.

Similarly, 10 ml of water representative sample was mixed with 90 ml of a sterilized peptone buffer and serially diluted up to 10^−7^. The bacterial count was expressed as log CFU ml^−1^ for water and fish samples. The total bacterial count was enumerated on a plate count agar (PCA) previously supplemented with 100 μl of each dilution (fish and water samples), then incubated at 37°C for 1 day (Saad et al., 2021), while the *Aeromonas* count was calculated on an *Aeromonas* agar medium after 1 day of incubation at 37°C (Sheir et al., 2020). The *Aeromonas* spp., colonies were distinguished by dark green with black-centered colonies.

### Statistical analysis

An ANOVA test was used to distinguish the significant differences between sample means at a probability level (*p* ≤ 0.05). The LSD test determined significant differences between means. All statistical calculations were performed with SPSS 20 for Windows.

## Results

### Isolation, screening, and identification of Se-resistant bacterial isolate

A total of 35 bacterial isolates were isolated from soil samples using PCA plates supplemented with sodium selenite (1 mM), 5 of which survived at a higher concentration (4 mM), with codes AS12, AS26, AS37, AS43, and AS66. One of them, AS12, could tolerate 5 mM sodium selenite and was considered a Se-resistant bacterium.

No colonies occurred at sodium selenite (6 mM). Based on the morphological, physiological, and biochemical tests in Bergey’s Manual, this isolate was aerobic, gram-positive, motile, long-rod, and spore-forming under a light microscope, which revealed that this bacterium is similar to the *Bacillus* species. Subsequently, the isolate was nominated as *Bacillus subtilis* AS12 based on MALDI TOF Mass Spectrometry.

Maximum similarity (99%) of the obtained isolate (*Bacillus subtitles* AS12) was established by the MALDI-TOF score to *Bacillus subtilis* DSM 5552 DSM. *B. subtilis* is an assemblage of closely-related species that cannot be easily differentiated based on phenotypic characteristics. In recent years, it has been disclosed that the identification of the 16S rRNA using gene-based phylogenetic analysis seems impossible because of the highly conserved nature of the gene.

As an alternative approach, the phylogenetic analysis of protein-coding loci can be used to identify and characterize the *Bacillus* species. MALDI-TOF MS identified the *B. subtilis* isolate as DSM 5552 DSM, with a score of 2.13 and NCBI identifier 1,432. According to GenBank Database BLAST results, the *B. subtilis* DSM 5552 DSM was registered based on the small acid-soluble spore protein gamma-type (sspE) gene as a complementary identification method of bacteria.

Our phylogenetic analysis was performed using the neighbor-joining method based on existing data of the sspE gene. Results revealed that the *B. subtilis* DSM 5552 DSM isolate is closely related to newly-isolated bacteria (*Bacillus subtilis* AS12), as shown in Supplementary Figure S1.

### Optimization of the physiochemical parameters for Bio-SeNPs

**Figure 1:**
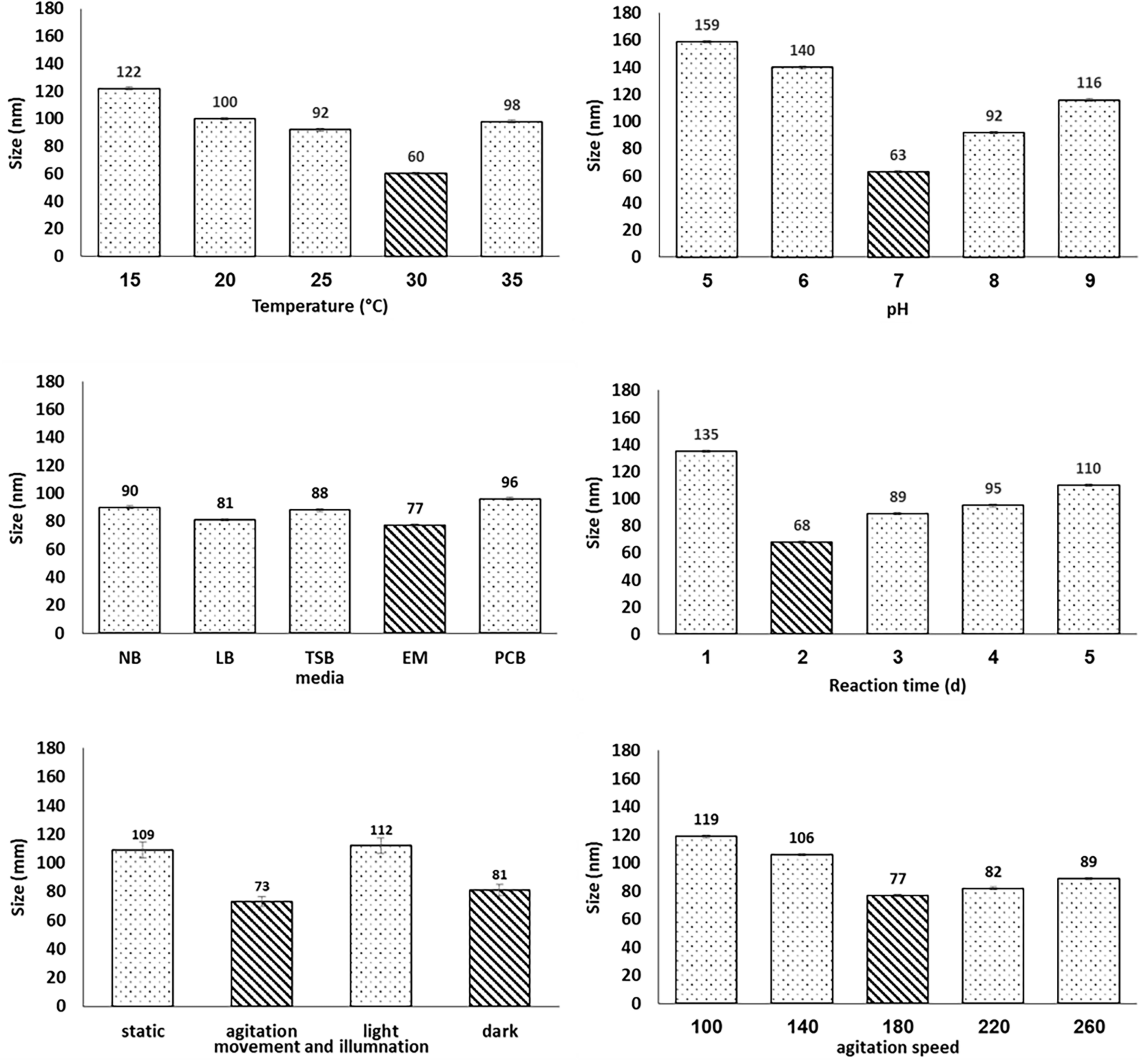
Optimization of the physiochemical parameters for fabricating by *Bacillus subtilis* AS12. Data are presented as means ± SE.

Figure 1 shows the optimal conditions for Bio-SeNPs fabrication by *Bacillus subtilis* AS12. The SeNPs with small size and high yield were obtained when the bacteria were inoculated on EM medium, and its supernatant was mixed with Na_2_SeO_3_ at pH 7. The mixture was incubated at 30°C for 2 days in a shaking incubator with an agitation speed of 180 rpm in the dark condition. These optimum conditions achieved SeNPs sizes ranging from 60 to 81 nm.

### Chemical qualification of Se in Bio-SeNPs

**Figure 2:**
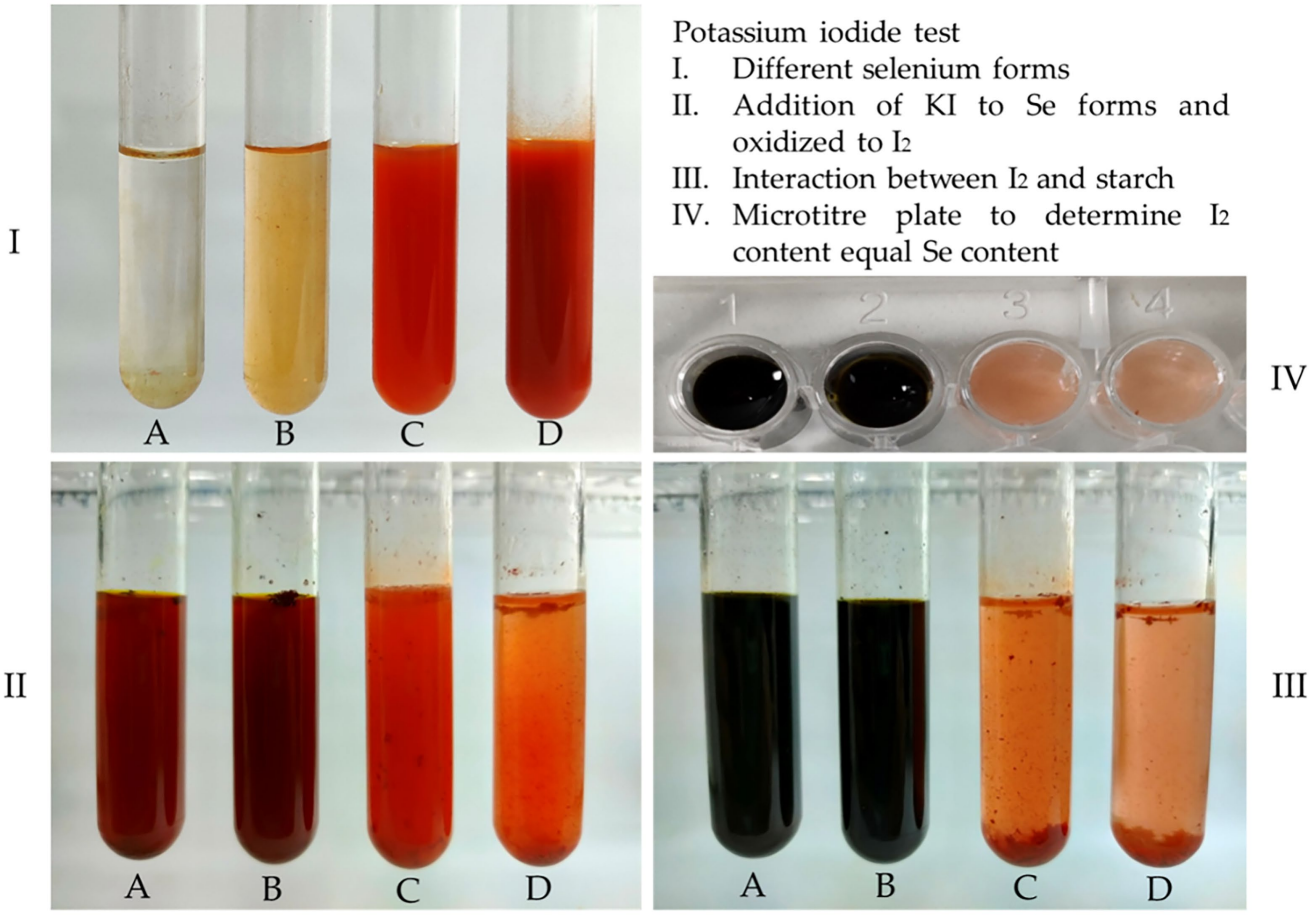
Qualitative identification of Selenium status in bio-selenium nanoparticles [BioSeNPs], obtained after 2d and 3d incubation with bacterial supernatant (C and D), as compared to two different controls:) Sodium selenite **(A)** and Sodium selenate **(B)**. I= The original selenium solutions. II= (I) + KI. III= (II) + Starch, IV= Microtiter plate to determine I_2_ content equal Se content.

Figure 2 shows the qualitative and quantitative colorimetric tests that evidenced the reduction of Na_2_SeO_3_ (Se^+4^) to the elemental Se form (Se0) in the Bio-SeNPs. The Tube-Series (No. 1) shows the control tubes (A) and (B) with transparent solutions of sodium selenite and sodium selenate. On the other hand, tubes (C) and (D), which contained the substrate sodium selenite and were incubated with the supernatant of *Bacillus subtilis* AS12 for 2 and 3 days, respectively, produced brick-red dispersions, primarily referring to elemental Se.

Therefore, it can be concluded that an incubation time between 2 and 3 days were sufficient to reduce sodium selenite into elemental Se under the metabolic action of *Bacillus subtilis* AS12. It was further evidenced by adding potassium iodide (KI) in an acidic medium to the tubes in series I, producing series II. The tubes in series (No. 2) showed that the control tubes (A and B) released I_2_, which could turn iodide into iodine because the Se was in its most oxidizing form. In contrast, tubes C and D had the uncharged form of Se. They did not have any sodium selenite leftover or any form of Se available to oxidize.

Thus, the total original amount of this Se form selenite (Se^+4^) may have transformed into elemental Se (Se0), which cannot oxidize iodide into iodine. However, since all the tubes in series II were brown, which might originate from iodine or the elemental Se, it was necessary to distinguish them. This distinguishing was done by interacting them with the starch solution to series II, resulting in tube series III. Tubes A and B of the series (No. 3) produced a blue color, confirming the release of iodine. At the same time, tubes C and D did not change color, confirming that no iodine was released and that the brick red color is solely due to the presence of the formed elemental Se (Se0) produced under the action of *Bacillus subtilis* AS12.

The inability of the Bio-Se-nanoparticles (tubes C and D) to oxidize KI into I_2_ implies that Se has changed its oxidation state from the original high values (selenite^+4^) into metallic Se (oxidation state equals zero). Alternately, the quantitative assessment of Se content in the nanoparticles recorded 4.83 mg/L, indicating the complete transformation of sodium selenite to Se^0^ in Bio-SeNPs under the action of *Bacillus subtilis* AS12.

### Characterization of biological se nanoparticles

**Figure 3:**
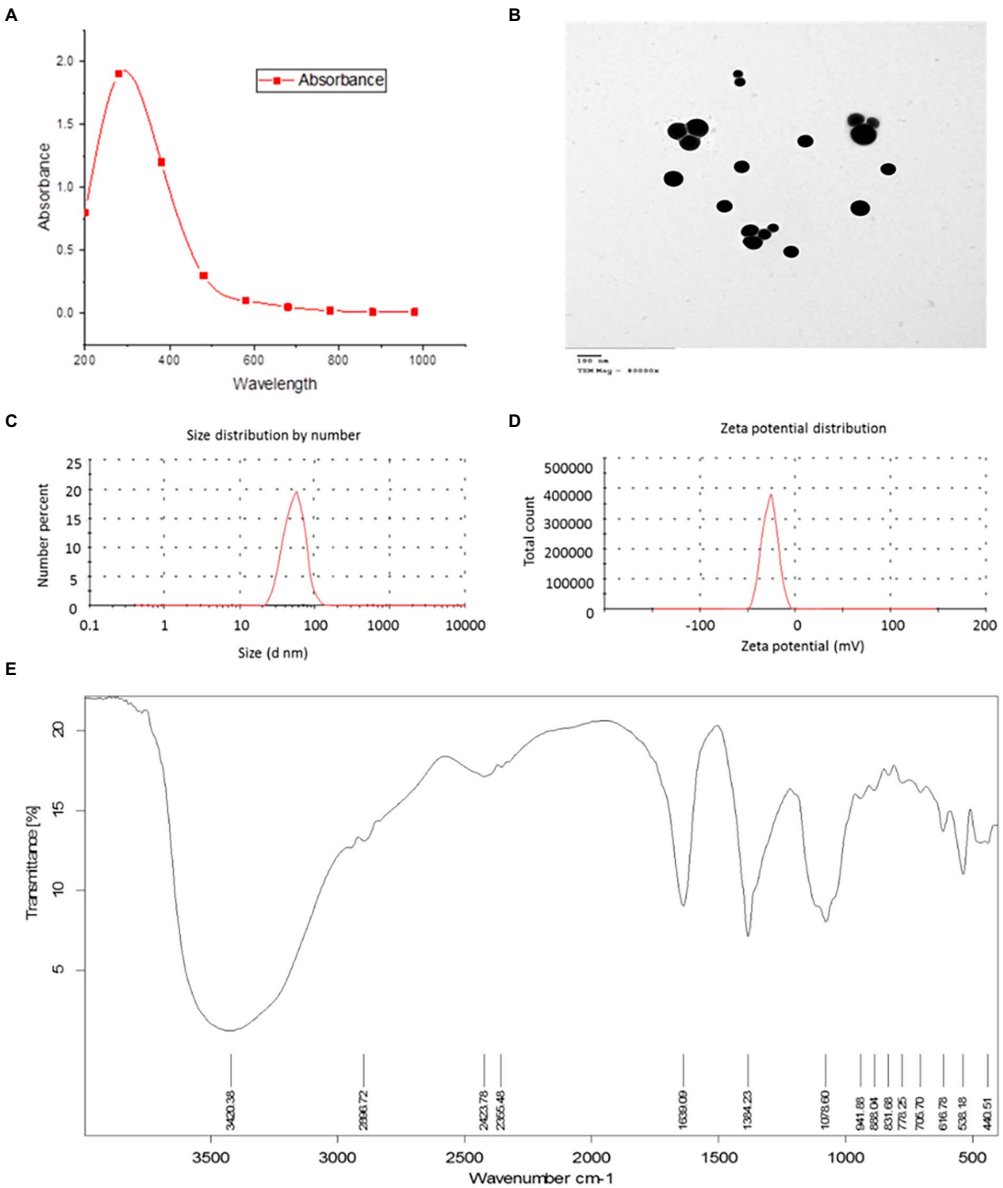
Characterization of biosynthesized selenium nanoparticles (BioSeNPs) synthesized by *Bacillus subtilis* AS12. **(A)** U.V. absorbance at 280 nm; **(B)** the size of 25–67 nm and its spherical shape in a transmission electron micropscopic image; **(C)** average size of 77.78 nm by Zeta sizer; **(D)** surface net negative charge by Zeta potential (−26.4); and **(E)** Fourier transform-infrared (FTIR) spectroscopy.

The color change from colorless to red when adding *Bacillus subtilis* AS12 pellets to the EM supplemented with Na_2_SeO_3_ solution indicates the biotransformation of Na_2_SeO_3_ into Bio-SeNPs. The UV–VIS spectroscopy, revealing the product’s absorption spectrum at 280 nm (Figure 3A), further confirms this transformation observation.

The TEM image (Figure 3B) of the produced Bio-SeNPs revealed a spherical shape with an average size in the range of 25–85 nm. SEM image of Bio-SeNPs showed in Supplementary Figure S2C. The FTIR of *Bacillus subtilis* AS12 pellets detected 9 bands (Supplementary Figure S3), while 15 bands between 3,420.38 and 440.51 cm^−1^ appeared in the FTIR spectrum of Bio-SeNPs (Figure 3E). These bands refer to the presence of various functional groups surrounding the SeNPs, e.g., alcohol, primary and secondary amines, amides, and phenols. The strong, broad peak at 3,420.38 cm^−1^ refers to the O–H and N–H groups, while the peak at 2,896.72 cm^−1^ refers to the CH group. Bands appearing at 2,423.78 and 2,355.48 cm^−1^ refer to the ester group, while bands at 1,639.09 and 1,384.23 cm^−1^ indicate alkenes and aromatics, respectively. The peak at 1,078.60 cm^−1^ indicated the presence of SO compounds, while the peaks at the range of 941.88–538.18 cm^−1^ referred to aromatic C–H, C﹦C, and halide compounds.

**Figure 4:**
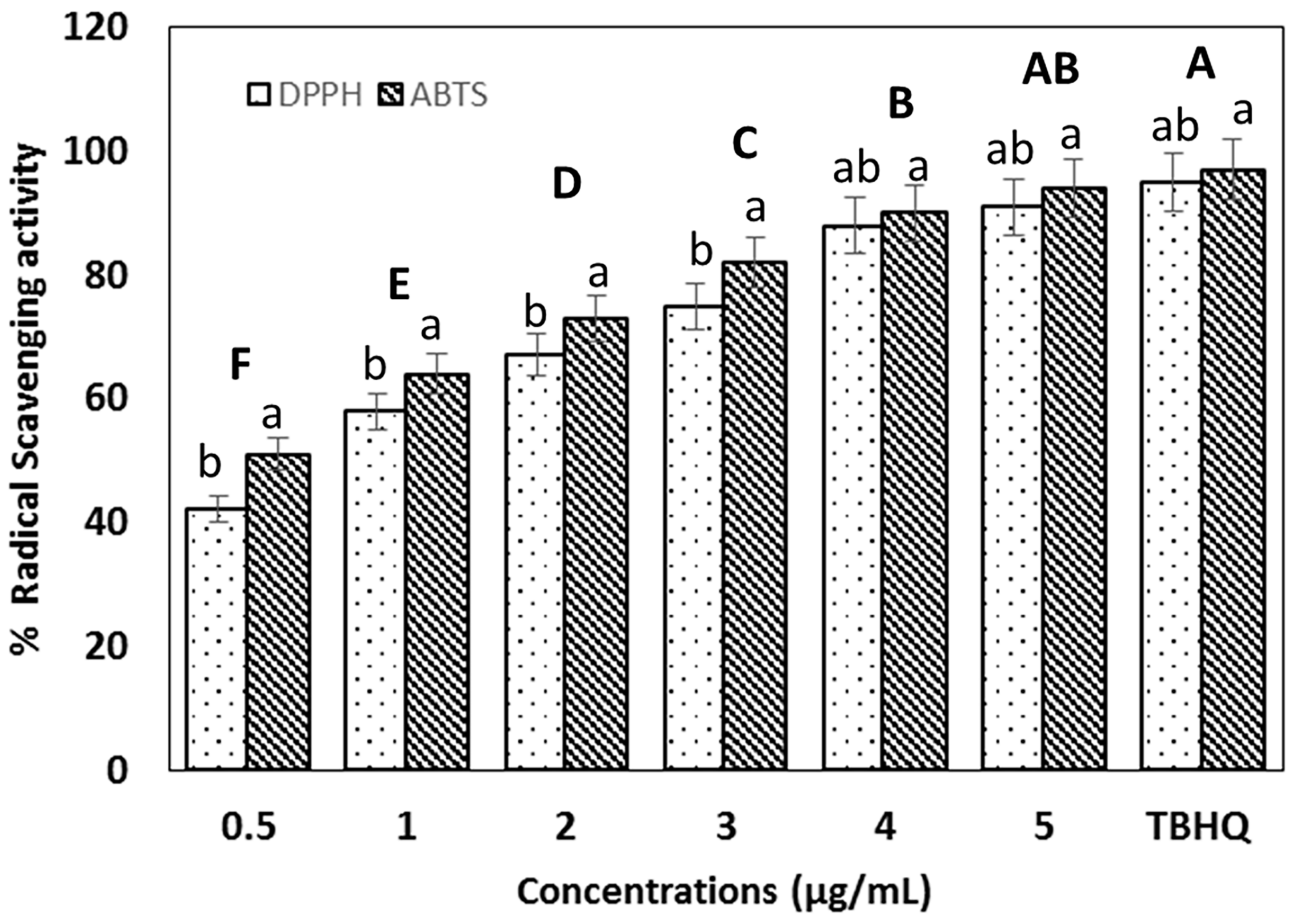
Radical scavenging activity of biosynthesized selenium nanoparticles (BioSeNPs) at different concentrations (0.5–5 μg ml^−1^) against ABTS and DPPH radicals after 30 min. of incubation, as compared to tret-butyl hydroquinone (TBHQ 5 μg ml^−1^). Data are presented as means ± SE. Bold uppercase letters indicate the significant differences between concentrations as compared to TBHQ, while lowercase letters indicate significant differences between DPPH and ABTS subjected to SeNPs and TBHQ.

Dynamic Light Scattering (DLS) is a technique used to study the behavior of nanoparticles in suspension in terms of size and charge. Figure 3C,D shows the DLS analysis of the stable Bio-SeNPs, indicating an exact size of 77.47 nm (Figure 4C), with a net negative charge of −26.33 mV (Figure 3D). These results confirm that the biotransformation of Bio-SeNPs was mono-dispersed.

XPS image confirming the production of elemental selenium founded in Supplementary Figure S2A XRD image in Supplementary Figure S2B revealed that Bio-SeNPs were in crystalline nature as evidenced by the peaks at 2θ value of 30° with stronger intensity of peaks for plane (101).

### Biological activity of Bio-SeNPs

#### Antioxidant activity

Figure 4 shows that Bio-SeNPs exhibited considerable ABTS+ and DPPḢ scavenging activity comparable to the synthetic antioxidant TBHQ (5 μg ml^−1^). The SC50 against DPPḢ and ABTS+ was 0.5 μg ml^−1^ and 1 μg ml^−1^, respectively. Antioxidant activity was significantly increased in a concentration-dependent manner. The Bio-SeNPs (5 μg ml^−1^) scavenged 91 and 94% of DPPḢ and ABTS+ compared to 95 and 97% in the case of TBHQ, respectively.

**Figure 5:**
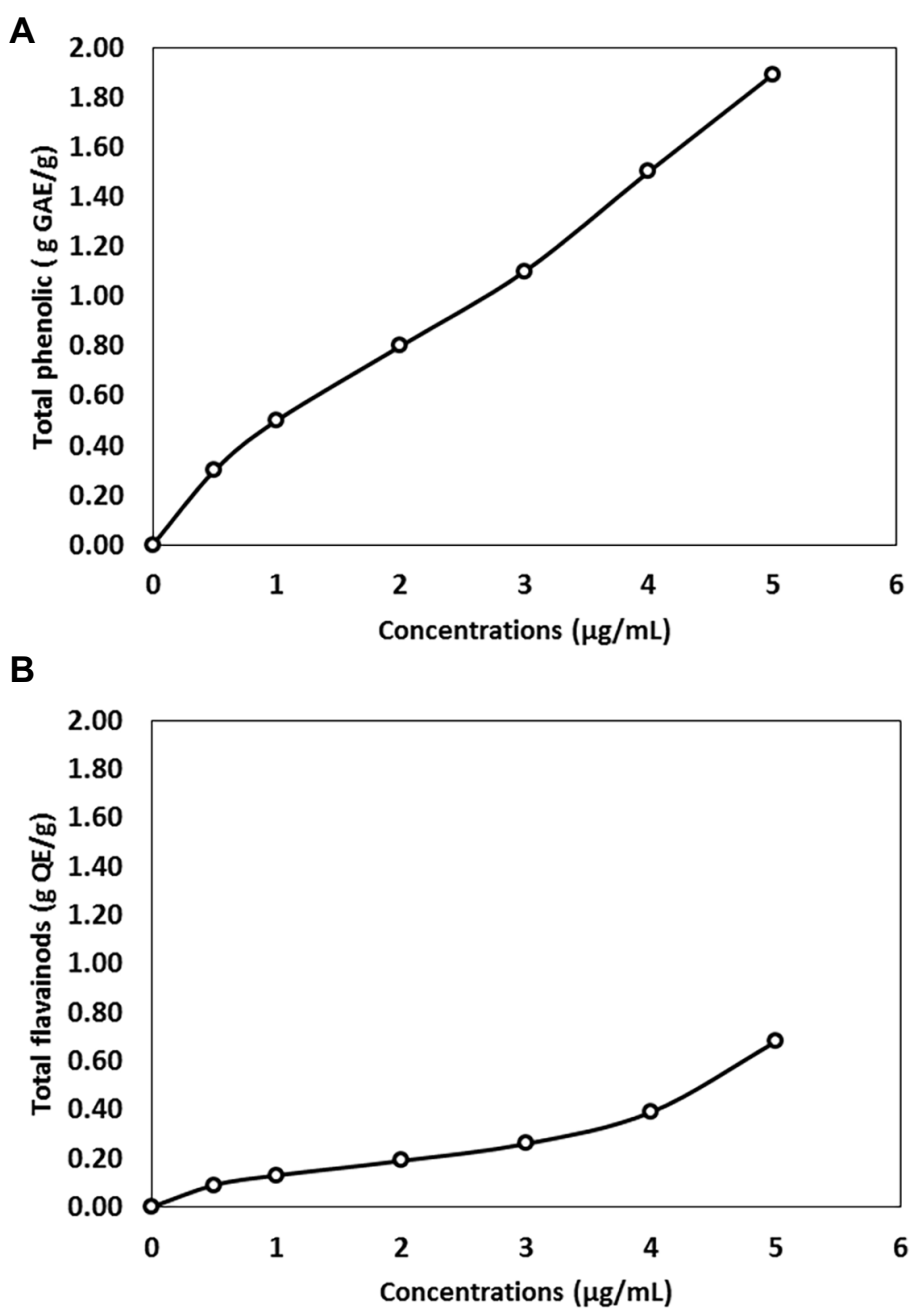
**(A)** Total phenolic content expressed as gallic acid equivalent (GAE) in biosynthesized selenium nanoparticles (BioSeNPs) suspension, fabricated by *Bacillus subtilis* AS12. **(B)** Flavonoids content expressed as Quercetin quercetin equivalent in BioSeNPs suspension fabricated by *Bacillus subtilis* AS12.

The phenolic and flavonoid content in Bio-SeNPs is presented in Figures 5A,B. The antioxidant activity of nanoparticles depended on the polyphenol content, which significantly increased concentration-dependent. The Bio-SeNPs (5 μg ml^−1^) have a total phenolic and flavonoids of 1.9 g/g and 0.7 g/g GAE and QE, respectively.

#### Antibacterial activity

**Figure 6:**
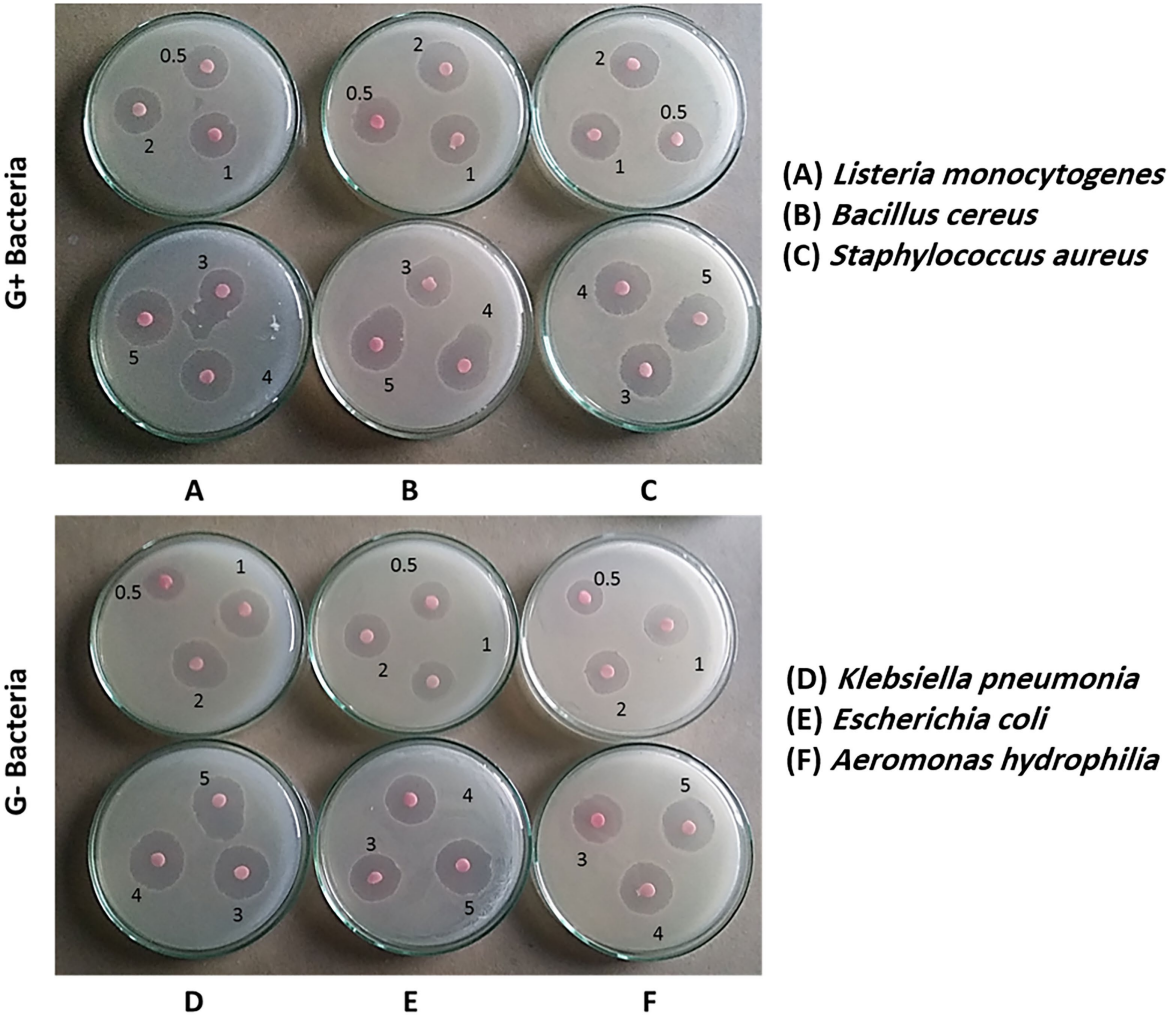
Antibacterial activity of biosynthesized selenium nanoparticles (BioSeNPs) suspensions (0.5–5.0 μg ml^−1^) fabricated by *Bacillus subtilis* AS12 against pathogenic Gram-positive bacteria (G+ bacteria) and Gram-negative bacteria (G− bacteria).

The inhibition zone diameters (IZDs) in Table 1 and Figure 6 show that Bio-SeNPs can kill bacteria. The IDZs increased significantly (*p* ≤ 0.05) with increasing Bio-SeNPs concentrations in the 0.5–5 μg ml^−1^ range. The IDZs ranged from 9 to 30 mm. *Staphylococcus aureus* and *Klebsiella pneumoniae* were the most sensitive G+ and G− bacteria to Bio-SeNPs (5 μg ml^−1^) with 30 mm and 24 mm, respectively.

**Table 1:** The inhibition zones diameters (IZDs) of the biosynthesized selenium nanoparticles (BioSeNPs) with minimum inhibitory concentration (MIC) and minimum bactericidal concentration (MBC) levels against certain Gram positive (G+) and Gram negative (G-) pathogenic bacteria.

Bacteria		SeNPs (μg ml^−1^) / IZDs (mm)						MIC	MBC
		0.5	1	2	3	4	5		
G+	*Listeria monocytogenes*	13bc	16b	18bc	20c	22c	25c	0.35b	0.65b
	*Bacillus cereus*	14b	17ab	19b	21b	24b	27b	0.30bc	0.55bc
	*Staphylococcus aureus*	16a	18a	21a	25a	27a	30a	0.25c	0.45c
G-	*Klebsiella pneumonia*	12c	14c	17c	20c	22c	24d	0.35b	0.65b
	*Escherichia coli*	10d	13 cd	15d	18d	20d	22e	0.40ab	0.75ab
	*Aeromonas hydrophilia*	9e	11d	14e	16e	18e	21f	0.45a	0.85a

Inhibition zones diameters (IZDs). Means in the same column with lowercase different letters are significantly different at *p* ≤ 0.05.

However, *Listeria monocytogenes* and *Aeromonas hydrophilia* were the most resistant G+ and G− bacteria to Bio-SeNPs (5 μg ml^−1^), recoding lower inhibition zones of 25 mm and 21 mm, respectively. The plates’ images in Figure 6 supported the results in Table 1. Generally, all tested bacteria were inhibited by Bio-SeNPs MIC in the range of (0.25–0.45 μg ml^−1^), and completely killed by Bio-SeNPs MBC (0.45–0.85 μg ml^−1^).

### Biological experiment on Nile tilapia fish exposed to Hg and Cd, and treated with Bio-SeNPs

#### Growth performance parameters

Adding the Bio-SeNPs to normal living fish, i.e., without subjection to heavy metal (group 1), improved the growth performance of fish (Table 2), meaning they exhibited weight gain and specific growth rate increases with all levels of nanoparticles. The treated fish with Bio-SeNPs levels (3–5 μg ml^−1^) gained weight in the 36–52% range, with a specific growth rate (SGR) of 40% higher than the control. At the same time, FCR was reduced, inferring improved food conversion. Therefore, using these Bio-SeNPs for normal living fish can enhance their growth performance. In parallel, survival in the Bio-SeNPs-treated fish was not negatively affected, supporting these nanoparticles’ safe and beneficial use.

**Table 2:** Growth performance of Nile tilapia (*Oreochromis niloticus*) fish subjected to heavy metals, mercury (Hg) and cadmium (Cd), and treated with biosynthesized selenium nanoparticles (BioSeNPs) during the 10-week experimental period.

Group	Subgroups	Cd + Hg(μg ml^−1^)	BioSeNPs(μg ml^−1^)	IW(g)	FW(g)	WG(g)	SGR (%/day)	TFI(g /fish)	FCR	SR (%)
1	1Z	–	–	20.0a	45.0d	25.0d	0.5b	35.0d	4.8ab	100.0a
	1A	–	3	20.5a	55.0c	34.5c	0.7a	51.0bc	4.6b	100
	1B	–	4	19.8b	57.0b	37.2b	0.7a	52.0b	4.0d	100
	1C	–	5	20.0a	58.0a	38.0a	0.7a	54.0a	4.2d	100
2	2Z	0.5 + 0.5	–	19.0b	47.0d	27.0 cd	0.59c	41.0 cd	4.7ab	91.0d
	2A	0.5 + 0.5	3	19.0b	52.0 cd	33.0c	0.6b	48.0c	4.3c	94.3c
	2B	0.5 + 0.5	4	19.5b	54.0c	34.5c	0.6b	50.0bc	4.5b	94.7b
	2C	0.5 + 0.5	5	20.0a	55.0c	35.0bc	0.7a	52.0v	4.6b	95.0b
3	3Z	1.0 + 1.0	--	19.4b	48.1d	28.7 cd	0.52c	47.0c	5.0a	89.9e
	3A	1.0 + 1.0	3	20.4a	51.3 cd	30.9 cd	0.6b	46.7 cd	4.4bc	92.8d
	3B	1.0 + 1.0	4	19.2b	52.5 cd	33.3c	0.6b	48.0c	4.5b	93.5c
	3C	1.0 + 1.0	5	19.5b	54.0c	34.5c	0.6b	49.0c	4.8ab	93.0c

Means in the same column with lowercase different letters are significantly different at *p* ≤ 0.05. IW, initial weight; FW, final weight; WG, weight gain; SGR, specific growth rate; TFI, total feed intake; FCR, feed conversion ratio; SR, survival rate.

Alternatively, exposing heavy metals, i.e., Hg and Cd mixtures at two levels (0.5 and 1 μg ml^−1^) in Nile tilapia (*Oreochromis niloticus*) media significantly influenced (*p* ≤ 0.05) the growth performance parameters. Weight gain improved (*p* ≤ 0.05) ≈ 0.0299 by ~8% and ~15%, and SGR was (*p* ≤ 0.05) ≈ 0.0302 increased by only 18 and 4%. However, the survival rate was decreased (*p* ≤ 0.05) ≈ 0.0385 by 9 and 10% in fish subjected to the first and second levels of heavy metals, respectively. FCR values fluctuated slightly with heavy metal additions in the two doses.

On the other hand, the water application of Bio-SeNPs at three levels (3, 4, and 5 μg ml^−1^) revealed greater positive effects on the growth performance of Nile tilapia in a concentration-dependent manner, consequently mitigating the negative effects of heavy metals. Weight gain in the second group (2A, 2B, and 2C) increased in the 22–30% range as compared to the respective control (2Z), and saw an ~8–20% increase in weight gain in the third group (3A, 3B, and 3C) compared to 3Z by the addition of Bio-SeNPs. Similarly, SGR was enhanced in treated fish in group 2 by 2–19%, compared to a 15% relative increase in group 3 in response to the 3 doses of the Bio SeNPs. Here, the survival rate improved by ~3–4% in the 2 groups compared to their respective non-treated controls.

#### Serum biochemical parameters

Table 3 presents the serum biochemical parameters of Nile tilapia (*Oreochromis niloticus*) fish both subjected and not subjected to the Hg and Cd mixture, at two levels in the water, 0.5 + 0.5 μg ml^−1^ and 1 + 1 μg ml^−1^, respectively. The Bio-SeNPs significantly reduced liver function marker levels (ALT and AST) in the heavy metal-free water and enhanced the innate immune responses, GH level, the male hormone, and XX hormones, but reduced both the F hormone P4 level and cortisol levels.

**Table 3:** Serum biochemical parameters, immune activity and hormonal levels of Nile tilapia (*Oreochromis niloticus*) fish subjected to heavy metals, mercury (Hg) and cadmium (Cd), and treated with the biosynthesized selenium nanoparticles (BioSeNPs) during the 10-week experimental period.

Group	Subgroups	Cd + Hg(μg ml^−1^)	BioSeNPs(μg ml^−1^)	Liver function			Innate immune response				Hormones					Cortisol(μg ml^−1^)
				NBT							FSH	T		P4	GH	
				Glucose (mg dl^−1^)	ALT (U/l)	AST (U/l)	(Abs)	LYZ (μg ml^−1^)	Ig (μg ml^−1^)	TP (mg dl^−1^)	(nIU ml^−1^)	(ng ml^−1^)		(ng ml^−1^)		
											M	F	XX			
1	1Z	–	–	103.00d	55.00c	130.00b	0.40e	1.30e	22.00c	46.00 cd	0.30d	0.95a	2.50e	0.90a	0.50e	3.70c
	1A	–	3	99.00e	49.00d	120.00c	0.60c	1.50c	30.00ab	60.00bc	0.50b	0.46e	2.90b	0.50d	0.67c	2.69e
	1B	–	4	96.00f	45.00e	115.00d	0.67b	1.60b	28.00b	61.30b	0.48b	0.43f	2.90b	0.47e	0.71b	2.45f
	1C	–	5	94.20f	43.30e	107.00e	0.85a	1.80a	31.00a	63.00a	0.55a	0.41f	3.10a	0.44f	0.80a	1.92 h
2	2Z	0.5 + 0.5	–	119.00b	76.00b	148.00ab	0.30f	1.09 g	18.00d	40.00d	0.25e	0.80b	2.35f	0.60b	0.55e	7.70b
	2A	0.5 + 0.5	3	108.00c	51.00c	130.00b	0.50d	1.30e	24.00c	51.00c	0.45c	0.60d	2.70c	0.56c	0.60d	2.80d
	2B	0.5 + 0.5	4	105.00d	48.00d	125.00c	0.49d	1.40d	25.60c	53.00c	0.48b	0.57de	2.78c	0.52d	0.69c	2.71d
	2C	0.5 + 0.5	5	99.00e	46.00d	119.00d	0.67b	1.53c	27.30b	54.40c	0.51b	0.52e	2.81c	0.49e	0.72b	2.11 g
3	3Z	1.0 + 1.0	–	125.00a	80.00a	150.00a	0.20 g	1.00 g	16.00d	38.00e	0.23e	0.70c	2.2	0.40f	0.60d	9.80a
	3A	1.0 + 1.0	3	110.00c	52.00c	135.00b	0.32f	1.20f	23.70c	48.00 cd	0.39d	0.72c	2.56d	0.58b	0.58e	3.10c
	3B	1.0 + 1.0	4	107.00c	50.00c	129.00c	0.31f	1.28e	24.60c	49.50 cd	0.41c	0.69 cd	2.62d	0.54c	0.61d	2.80d
	3C	1.0 + 1.0	5	102.00d	48.30d	121.00d	0.49d	1.30e	25.20c	51.00c	0.43c	0.66d	2.70c	0.51d	0.66c	2.50e

Means in the same column with lowercase different letters are significantly different at *p* ≤ 0.05. ALT, alanine transaminase; AST, aspartate transaminase; NBT, nitrobluetetrazolium; LYZ, lysozyme; Ig, immunoglobulin; TP, total protein; FSH, follicle-stimulating hormone; T, testosterone; M, male; F, female; XX, intersex; P4, progesterone; GH, growth hormone.

Considerable biological changes occurred from the subjection of fish to heavy metals. Glucose levels increased by 16 and 21% in fish subjected to the 2 heavy metals (2Z and 3Z) levels over the untreated control (1Z). Heavy metals adversely affected liver functions, raising ALT and AST enzymes by 38 and 45% and by 14 and 15% in 2Z and 3Z over the control (1Z), respectively. The innate immunity, responses, and growth hormones were moderately reduced by fish subjected to heavy metals in a heavy metal concentration manner. Cortisol levels in fish exposed to heavy metals (2Z and 3Z) went up by 108 and 165%, respectively, compared to the control group (1Z) fish. These changes were proportional to the concentration of the metals.

Alternately, treating fish subjected to heavy metals with Bio-SeNPs at three graded levels (3, 4, and 5 μg ml^−1^) efficiently reversed the heavy metals’ harmful effects in a concentration-dependent manner. The second concentration of Bio-SeNPs (4 μg ml^−1^) can mostly eliminate the adverse effects of heavy metals exposed at a medium dose (0.5 μg ml^−1^ and 0.5 μg ml^−1^ of Cd and Hg), while the highest concentration of Bio-SeNPs (5 μg ml^−1^) was required to eliminate adverse effects originating from high heavy metal doses (1 μg ml^−1^ and 1 μg ml^−1^ of Cd and Hg). Therefore, applying Bio-SeNPs in the rising environment can efficiently and effectively alleviate the adverse effects of heavy metals on the serum biochemical parameters of fish.

#### Behavioral patterns

Table 4 shows the behavioral patterns of fish exposed to heavy metals throughout a 10-week experiment. Initially, the sole presence of Bio-SeNPs in the water enhanced the feeding time and frequency in a concentration-dependent manner, with the maximum relative increases amounting to 60 and 66% in feeding time and frequency for 5 μg ml^−1^ over the untreated fish.

**Table 4:** Behavioral patterns of Nile tilapia (*Oreochromis niloticus*) fish subjected to the heavy metals, mercury (Hg) and cadmium (Cd), and treated with biosynthesized selenium nanoparticles (BioSeNPs) during the 10 week experimental period.

Groups	Subgroups	Cd + Hg(μg ml^−1^)	BioSeNPs(μg ml^−1^)	Feeding time (sec)	Feeding frequency	Foraging time (sec)	Foraging frequency	Surfacing time (sec)	Surfacing frequency	Swimming time (sec)	Swimming frequency	Resting time (sec)	Resting frequency	Arousal time (sec)	Arousal frequency	Aggression time (sec)
1	1Z	–	–	120.0 g	6.0e	140.0a	4.0c	220.0e	9.2ab	1180.0e	25.0c	400.0b	7.0c	900.0f	23.0d	190.0a
	1A	–	3	160.0d	7.2c	130.0d	3.8 cd	220.0e	8.5b	1255.0d	25.4c	405.0b	7.5c	1125.0c	24.0c	65.0d
	1B	–	4	175.2c	8.9b	124.0e	3.1d	219.0f	7.3c	1354.0c	26.9b	410.0ab	8.4ab	1156.0b	25.1b	42.0f
	1C	–	5	192.0a	10.0a	120.0f	2.5e	221.0e	6.8d	1500.0a	28.0a	420.0a	8.8a	1200.0a	29.0a	25.0 g
2	2Z	0.5 + 0.5	–	104.0 h	6.4d	138.0b	5.1ab	250.0ab	9.1ab	1104.0f	24.0d	300.0e	6.5d	996.0e	23.6d	135.0b
	2A	0.5 + 0.5	3	145.0e	6.1e	135.0c	4.8b	231.0c	8.2bc	1198.0e	24.0d	320.0d	7.0c	1025.0d	24.0c	70.0d
	2B	0.5 + 0.5	4	167.0 cd	7.2c	130.0d	4.0c	226.0d	7.7c	1289.0d	25.1c	340.0c	7.5c	1105.0c	24.2c	56.0e
	2C	0.5 + 0.5	5	185.0b	8.2bc	128.0e	3.2d	220.0e	7.0d	1405.0b	26.0b	380.0c	8.0b	1150.0b	25.0b	30.0 g
3	3Z	1.0 + 1.0	–	78.0i	6.7d	141.0a	5.5a	255.0a	9.7a	1090.0f	24.0d	290.0e	6.0e	998.0e	23.0d	90.0c
	3A	1.0 + 1.0	3	135.0f	6.0e	137.0b	4.5b	240.0b	8.9b	1150.0e	24.7c	310.0d	6.4e	1011.0d	24.2c	87.0 cd
	3B	1.0 + 1.0	4	151.0d	6.4d	131.0c	4.1c	235.0c	8.2bc	1240.0d	25.0c	325.0d	6.9d	1089.0d	24.8c	67.0d
	3C	1.0 + 1.0	5	167.0 cd	7.0c	129.0e	3.9 cd	230.0c	7.9c	1350.0c	25.8c	350.0c	7.5c	1120.0c	26.0b	42.0f

Means in the same column with lowercase different letters are significantly different at *p* ≤ 0.05.

Bio-SeNPs treatments led to a moderate increase in swimming time and frequency but only a slight increase in resting time and frequency. Moderate decreases accompanied these increases in both foraging time and frequency. While surfacing time was not affected, Bio-SeNPs moderately reduced the surfacing frequency. Arousal time was increased while aggregation time was greatly reduced, with a maximum reduction of 78% being the highest amount of Bio-SeNPs (5 μg ml^−1^).

A different trend was observed from the exposure to heavy metals (Cd + Hg). The feeding time decreased by ~13 and 35%, with the 2 levels of heavy metals, 0.5, and 1 mg L^−1^, respectively, while the feeding frequency was slightly increased (7 and 12%, respectively). Surfacing time increased by ~14–16% while surfacing frequency increased by ~5% to the high level of heavy metal exposure. Foraging time slightly fluctuated, but the foraging frequency was moderately increased by exposure to heavy metals.

Both swimming time and frequency were slightly reduced while resting time and frequency were only moderately reduced by exposure to heavy metals. Both levels of heavy metal exposure slightly increased arousal time and frequency. In contrast, aggression time was reduced by 29 and 53% at the first and second levels of heavy metal exposure, respectively.

Water application of Bio-SeNPs mitigated the adverse effects of Cd + Hg on the behavioral patterns of fish. For example, the decreasing effect of heavy metals on feeding time was encountered by an increasing effect in the 28–44% range in the group exposed to 0.5 + 0.5 μg ml^−1^ (Cd + Hg), and in the 42–53% range in the group exposed to 1 + 1 μg ml^−1^ (Cd + Hg), in response to (3–5 μg ml^−1^), respectively. The increase in foraging frequency induced by heavy metals was reversed by considerable reductions in the 6–37% and the 18–29% range in groups 2 and 3, respectively. Surfacing behavior decreased in the 10–23% range in group 2 and the 8–19% range in group 3 when treated with Bio-SeNPs (3–5 μg ml^−1^) compared to the untreated control.

The decreases in swimming time and resting time caused by exposure to heavy metals were considerably reversed when treated with Bio-SeNPs (3–5 μg ml^−1^). The observed increases in arousal time and frequencies in fish exposed to heavy metals were further enhanced by Bio-SeNPs (3–5 μg ml^−1^) in groups 2 and 3, with ranges of 3–13% and 1–11%, respectively. The reductions in the aggression time noticed when fish were exposed to heavy metals were also further reduced by treatment with Bio-SeNPs (3–5 μg ml^−1^).

### Heavy metal content in fish organs at experiment end

Table 5 presents the accumulation of Hg and Cd (μg/g) in fish organs, liver, intestines, gills, and kidney after 10 weeks of exposure to heavy metals and treatment with Bio-SeNPs concentrations of 3, 4, and 5 μg ml^−1^. Control fish in the free heavy metal water exhibited low levels of heavy metals. The presence of Bio-SeNPs further eliminated heavy metals to undetectable levels in fish organs. Subjecting fish to heavy metals in water resulted in massive levels of heavy metal accumulation in fish organs. Cadmium accumulation in exposed fish was 25–69 times the amount observed in organs of untreated fish (0.5 μg ml^−1^ Cd). At the same time, Hg accumulation was in the 38–128 times range of fish subjected to the first level of heavy metals in water (0.5 μg ml^−1^ Hg). When doubling the heavy metal exposure level (1 μg ml^−1^ for both Cd and Hg), the range of heavy metal accumulation in fish organs excelled to 32–170 times for Cd and 88–280 times for Hg.

**Table 5:** Content of heavy metals, mercury (Hg) and cadmium (Cd) in Nile tilapia (*Oreochromis niloticus*) fish organs (liver, intestine, gills and kidney) at the end of experiment (10 weeks), and the reducing effect of biosynthesized selenium nanoparticles (BioSeNPs).

Group	Subgroups	Cd + Hg(μg ml^−1^)	BioSeNPs(μg ml^−1^)	Hg (μg g^−1^)				Cd (μg g^−1^)			
				Fish organs							
				Liver	Intestine	Gills	Kidney	Liver	Intestine	Gills	Kidney
1	1Z	–	--	0.02 g	0.3e	0.01 g	0.1f	0.04 g	0.03f	0.01f	0.01e
	1A	–	3	ND	ND	ND	ND	ND	ND	ND	ND
	1B	–	4	ND	ND	ND	ND	ND	ND	ND	ND
	1C	–	5	ND	ND	ND	ND	ND	ND	ND	ND
2	2Z	0.5 + 0.5	–	0.75d	8.6bc	0.69c	2.55c	1.5c	3.85c	0.5c	0.62c
	2A	0.5 + 0.5	3	0.56e	3.2c	0.49d	1.89d	1.1d	2.4d	0.23d	0.46 cd
	2B	0.5 + 0.5	4	0.4ef	3.0c	0.32e	1.5de	0.98e	2.1e	0.16e	0.27d
	2C	0.5 + 0.5	5	0.1f	2.3d	0.1f	1.2e	0.65f	1.9e	0.09f	0.12e
3	3Z	1.0 + 1.0	--	1.9a	16a	1.7a	7.9a	3.5a	8.4a	1.1a	1.4a
	3A	1.0 + 1.0	3	1.5b	10b	1.4ab	3.9b	2.14b	4.2b	0.8b	0.98b
	3B	1.0 + 1.0	4	1.1c	8.9bc	1.0b	3.1bc	1.87c	3.7c	0.5c	0.7c
	3C	1.0 + 1.0	5	0.8d	5.8c	0.5d	2.3c	1.5c	2.6d	0.3c	0.5 cd

Means in the same column with lowercase different letters are significantly different at *p* ≤ 0.05.

Treating the polluted water with Bio-SeNPs (3, 4, and 5 μg ml^−1^) mitigated the accumulation of heavy metals in fish organs in a concentration-dependent manner. Treating the contaminated media with Bio-SeNPs at 5 μg/ml significantly reduced the heavy metal accumulation in group 2 (low level of heavy metal exposure) by 50–87% compared to untreated fish. Simultaneously, reducing the heavy metal accumulation by 57–73% in case of high levels of heavy metal exposure (group 3).

### Bacterial load in water and fish organs at experiment end

The levels of contaminating bacteria (total bacterial count and *Aeromonas* count) in the fish water media during the 10 weeks are shown in Table 6. Uncontaminated media contained a total bacterial count increasing from 5.4 Log CFU ml^−1^ in the first week to 6 Log CFU ml^−1^ in the tenth week. The total *Aeromonas* count increased from 2.9–3.9 Log CFU ml^−1^ for the same period. Including Bio-SeNPs (5 μg ml^−1^) in the uncontaminated water media at different concentrations (group 1) reduced the bacterial load in a concentration-dependent manner. Maximum reductions amounted to 27–35% and 36–42% in response to the highest level of Bio-SeNPs.

**Table 6:** Total bacterial and *Aeromonas* counts in fish farming water during the 10-weeks experimental period.

Groups	Subgroups	Cd + Hg	BioSeNPs	Total bacterial count				Total *Aeromonas* count			
				Log CFU ml^−1^							
		(μg ml^−1^)	(μg ml^−1^)	Experimental period (weeks)							
				1	4	8	10	1	4	8	10
1	1Z	–	–	5.4a	5.7a	5.9a	6.0a	2.9a	3.3a	3.7a	3.9a
	1A	–	3	4.1bc	4.3c	4.6d	4.9c	2.2c	2.4c	2.8c	3.0c
	1B	–	4	3.7 cd	4.0c	4.3d	4.6d	2.0d	2.1d	2.4d	2.8d
	1C	–	5	3.5d	3.7d	4.1d	4.4d	1.7d	1.9d	2.2d	2.5d
2	2Z	0.5 + 0.5	–	5.2a	5.3a	5.5ab	5.7ab	2.7ab	2.9ab	3.4ab	3.7ab
	2A	0.5 + 0.5	3	4.3b	4.5b	4.9c	5.0b	2.3c	2.5c	2.7c	3.0c
	2B	0.5 + 0.5	4	3.9c	4.1c	4.5d	4.8c	2.1c	2.3c	2.6c	2.8d
	2C	0.5 + 0.5	5	3.7 cd	3.9d	4.2d	4.4d	1.9d	2.1d	2.4d	2.6d
3	3Z	1.0 + 1.0	–	4.8b	5.0ab	5.2b	5.5b	2.4b	2.6b	3.1b	3.4b
	3A	1.0 + 1.0	3	4.5b	4.8b	5.0b	5.3b	2.5b	2.7b	3.0b	3.2b
	3B	1.0 + 1.0	4	4.0c	4.3c	4.8c	5.0b	2.3c	2.5c	2.8c	3.0c
	3C	1.0 + 1.0	5	3.8c	4.0c	4.6d	4.8c	2.1c	2.3c	2.6c	2.8d

Means in the same column with lowercase different letters are significantly different at *p* ≤ 0.05.

In heavy metal-contaminated water media, total bacterial counts were slightly reduced by ~4–7% and 8–12%, at the low and high levels of applied heavy metals, respectively, against relative increases in *Aeromonas* counts of 5–12% and 13–18%, respectively. Adding Bio-SeNPs (5 μg ml^−1^) to heavy metal-contaminated media further reduced the levels of bacterial counts (Table 6).

Bacteria levels in the media were reflected in the bacterial counts within fish organs, particularly those within direct contact with water, i.e., the skin, the gills, and the intestine (Table 7). These organs recorded high levels of total bacterial counts in the 7.5–8.2 Log CFU ml^−1^ range and 3.1–4.7 Log CFU ml^−1^ range of *Aeromonas* count. The addition of Bio-SeNPs (3–5 μg ml^−1^) reduced these levels by ~28–42% in both total bacterial and total *Aeromonas* counts.

**Table 7:** Total bacterial and *Aeromonas* counts in Nile talipa (*Oreochromis niloticus*) fish organs (skin, muscle, intestine and gills) after 10 weeks of treatment.

Groups	Subgroups	Cd + Hg(μg ml^−1^)	BioSeNPs(μg ml^−1^)	Total bacterial count Log CFU ml^−1^				Total *Aeromonas* count Log CFU ml^−1^			
				Fish organs							
				Skin	Muscle	Intestine	Gills	Skin	Muscle	Intestine	Gills
1	1Z	–	–	7.5a	5.9a	7.9a	8.2a	4.5a	3.2a	4.7a	5.1a
	1A	–	3	5.8d	4.0d	6.2d	6.7c	3.7c	2.5b	3.5c	3.9c
	1B	–	4	5.4de	3.7e	5.7e	6.3e	3.5c	2.3c	3.0d	3.5d
	1C	–	5	5.2e	3.4e	5.4e	5.9e	3.2d	1.9d	2.8d	3.2d
2	2Z	0.5 + 0.5	–	7.1ab	5.7ab	7.5ab	7.8b	4.2ab	3.1ab	4.3ab	4.7b
	2A	0.5 + 0.5	3	6.2c	4.5c	6.5c	6.9c	4.0b	2.7b	3.7c	3.9c
	2B	0.5 + 0.5	4	6.0c	4.3d	6.0d	6.5d	3.7c	2.3c	3.5c	3.7c
	2C	0.5 + 0.5	5	5.8d	4.0d	5.8e	6.3e	3.5c	2.0d	3.2d	3.5d
3	3Z	1.0 + 1.0	–	6.7b	5.1b	7.2b	7.3bc	4.0b	3.0ab	4.1b	4.3b
	3A	1.0 + 1.0	3	6.5b	4.8c	6.7c	7.1c	4.1b	2.9b	4.1b	4.0bc
	3B	1.0 + 1.0	4	6.2c	4.5c	6.5c	6.9c	3.9c	2.6b	3.8c	3.7c
	3C	1.0 + 1.0	5	6.0c	4.3d	6.2d	6.6d	3.7c	2.4c	3.6c	3.4d

Means in the same column with lowercase different letters are significantly different at *p* ≤ 0.05.

Exposure to high levels of heavy metals (Cd + Hg) significantly decreased the total bacterial count in fish organs within a 9–14% range and total *Aeromonas* within a 6–16% range over the respective controls. Combining Bio-SeNPs application with heavy metals further reduced the bacterial counts.

## Discussion

In this study, a selenium (Se)-resistant bacteria, *Bacillus subtilis* AS12, was isolated from the soil and identified by morphological, physiological, and biochemical tests following Bergey’s Manual (Wang et al., 2007). It was considered safe (GRAS) for this study. Further identification by MALDI-TOF qualified the isolate as *B. subtilis* DSM 5552 DSM. The high identity of 99.77–99.25% between *B. subtilis* DSM 5552 DSM and the other *B. subtilis* species showed that phylogenetic analysis based on MALDI-TOF MS results and NCBI identifiers could be a reliable, complementary, and alternative way to identify and classify *B. subtilis*. This observation can avoid the complicated differentiation of closely related *B. subtilis* based on 16S rRNA (Chun and Bae, 2000; Palmisano et al., 2001; Gatson et al., 2006).

To date, this is the first study chemically evidencing the complete transformation of Na₂SeO₃ (Se+4) into Bio-SeNPs (Se0) with *Bacillus subtilis* AS12, resulting in the conversion of all Se^+4^ to elemental Se, as quantitatively assessed at 4.83 mg/l in the final nanoparticles. The bacterial suspension has alkaloids, tannin, cinnamic acid, phenolic acid, monoterpenes, and secondary metabolites that can bind to the metallic core and help make Se nanoparticles with stable shapes and sizes (Javed et al., 2020).

*Bacillus* species have been reported as nanoparticle producers, namely, *B. mycoides* (Cremonini et al., 2016) and *B. pumilus* sp. BAB-3706 (Prasad et al., 2015), *B. licheniformis* (Sonkusre, 2020), and *B. amyloliquefaciens* SRB04 (Ashengroph and Hosseini, 2021). The Bio-SeNPs, produced by *Bacillus subtilis* AS12 in this study, were spherical, at 67 nm in diameter, with a − 26.33-mV negative charge, and rich in various bioactive natural compounds. The obtained results similar to other work with different species of *Bacillus*. For example, Pouri et al. (2017) synthesized spherical SeNPs with a size of 36 nm by *Bacillus cereus*; and Abdallah et al. (2019) fabricated spherical SeNPs, at 60–125 nm diameter and −30.6 negative charge by *Bacillus tropicus* Ism 2 (MK332444), isolated from soil polluted with industrial wastewater.

The prepared Bio-SeNPs showed considerable antioxidant activity to scavenge the ROS, which damages the DNA and protein. The scavenging power of Bio-SeNPs may be due to the wide surface area and the released Se. The potential action of the associated phenolic and flavonoid content derived from the components of the bacterial media, LB, and supernatant acted as reducing agents, which is required for nanoparticle production. Yeast extract is one of the medium components, which may be the source of several phenolic compounds (Vieira et al., 2016; El-Saadony et al., 2021d). In a similar study, *L. acidophilus* ML14 fabricates different Se nanoparticles (El-Saadony et al., 2021b), and (Abdel-Moneim et al., 2022) found that *Bacillus subtilis* AL43 exhibits antioxidant activity. These components may also contribute to the observed antibacterial activity of the NPs following Abdel-Shafi et al. (2019a).

Generally, all tested pathogenic bacteria were inhibited by Bio-SeNPs concentrations in the range of 250–450 ng ml^−1^ and entirely killed by 450–850 ng ml^−1^. These effective concentrations are vastly lower than previously reported native- or chemically-modified natural proteins, including soybean glycinin, chickpea protein (Sitohy et al., 2012; Osman et al., 2018), or esterified proteins (Sitohy and Osman, 2010). However, several nanoparticles, such as silver NP showing antibacterial activities are probably associated with harmful side effects (Huang et al., 2019; Li et al., 2020). Unlike other metals, Se is an essential element that can be administered as a green nanoparticle. Also, the antibacterial activity of the Bio-SeNPs decreased with their size. For example, SeNPs with 81 nm were more effective against *S. aureus* than SeNPs with a size of 124 nm (Huang et al., 2019).

Based on these results, it can be assumed that the small size of the Bio-SeNPs may explain why they are so effective as antibacterial. The low MIC values observed with Bio-SeNPs (250–450 ng ml^−1^) match and excel the values reported for *Penicillium chrysogenum*-mediated SeNPs 3.950–0.245 μg ml^−1^ (El-Sayyad et al., 2020), probably due to the different composition of each preparation.

The possible antimicrobial potential of Bio-SeNPs may occur through damaging the plasma membrane, which affects its functionality and integrity, or through altering the deoxyribonucleic acid replication, protein synthesis process, and food metabolism cycle (Mosallam et al., 2018; El-Gazzar and Ismail, 2020). In contrast to the positively charged antimicrobial basic proteins (Abdel-Shafi et al., 2016), nanoparticles carry negative charges on their outer surfaces. The positive charges were the main driving force for these basic proteins, which can distort the bacterial membranes through chemical electrostatic interactions with the negative charges on the cell membranes (Sitohy et al., 2011, 2013).

Accordingly, it is unlikely that the negatively charged NPs (−26.33 mV) will act in the same chemical way with negatively charged cell membranes. As a result, the possible interaction of Bio-SeNPs may go through physical and not chemical interactions. The potential interaction may start with repulsion between the similar negative charges of both NPs and the cell membrane, generating a random mechanical movement of the NPs in all directions and causing the collision of nanoparticles with other bacterial cells. This potential collision between the same NPs based on similar negative charges may double the collisional motions with bacterial cells, leading to accelerated cellular damage. Further, explain the strong antibacterial activity of NPs.

As rigid and strict structures generally characterize heavy metal nanoparticles (Khan et al., 2019), their collision with bacterial cells may be quite destructive to the bacterial cells. The collision force may depend on the rigidity of NPs and the speed of their random movement, which may depend on the NPs’ size. Thus, the medium size of Bio-SeNPs (~67 nm) may guarantee the high speed, thereby supporting its effectiveness. Thus, the nanoparticles’ speed and rigidity may contribute to the effectiveness of the physical antimicrobial action of NPs (Hashem et al., 2021; Elakraa et al., 2022; Hashem and Salem, 2022; Salem, 2022b).

The spherical shape of Bio-SeNPs may allow a multitude of simultaneous interactions with different bacterial cells, thus contributing to the effectiveness of the NPs and explaining the very low MIC values against different pathogenic bacteria. Once the outer cell membrane is distorted, the NPs can interact indiscriminately with any positively-charged cellular component, assuring the death of the entire bacteria. This physical mechanism of the NPs’ action may explain the relatively lower MIC values of Bio-SeNPs (250–450 ng ml^−1^) than the antimicrobial proteins 50–200 μg ml^−1^ (Abdel-Shafi et al., 2019b; Saad et al., 2021). This MIC level is also much lower than other synthetic NPs (Sitohy et al., 2021), showing that this green nanoparticle preparation is a better antimicrobial agent.

Several techniques remedy the presence of heavy metals in wastewater, e.g., electrolysis, adsorption, coagulation, flocculation, and membrane filtration (Chen et al., 2013; Zeng et al., 2015). However, the effectiveness and efficiency of these techniques are still low. Biogenic SeNPs have attracted attention in the last few decades and have been recommended as effective adsorbents for removing heavy metals from contaminated media (Nancharaiah and Lens, 2015). Additionally, Qi et al. (2021) reported that SeNPs scavenged the ROS induced by cadmium (Cd) contamination by inhibiting the activities of NADPH and glycolate oxidases. In addition, it has been reported that SeNPs can eliminate elemental mercury (Hg) from groundwater (Wang et al., 2018).

Selenium is essential to produce selenoproteins in the glutathione cycle. Consequently, the deficiency of Se in aquafeed causes malnutrition and loss in weight gain in various fish species (Kumar et al., 2020; Wang et al., 2021). Furthermore, biogenic SeNPs exhibited considerable antimicrobial potential against several pathogenic microorganisms in various fish (Khiralla and El-Deeb, 2015; Korde et al., 2020).

Bio-SeNPs in this study significantly enhanced growth performance in Nile tilapia fish by enhancing feed efficiency and increasing nutrient availability for optimal metabolic functions (Swain et al., 2019; Abd El-Kader et al., 2020; Dawood, 2021). Furthermore, the addition of Se enhances the gastrointestinal absorption capacity by enhancing protein utilization and improving the activity of digestive enzymes for properly utilizing food (Iqbal et al., 2020; Adineh et al., 2021), indicating the role of SeNPs as a growth promoter and safe additive for aquaculture without adverse side effects. Furthermore, the positive effect of SeNPs on the upregulation of the Insulin-like growth factor 1 (IGF-1) gene expression, enhancing the ghrelin hormone, and improving feed utilization can improve growth performance (Abd El-Kader et al., 2020; Jahanbakhshi et al., 2021).

In this regard, Çiçek et al. (2021) found that adding SeNPs to the diets of common carp, Nile tilapia, European seabass, red seabream, and yellowfin seabream led to a significant improvement in growth performance. Furthermore, Kumar et al. (2018) reported improving growth performance traits, e.g., WG, SGR, and PER values in striped catfish grown under high Pb concentration and high-temperature stress of 34°C, particularly when fish were delivered the SeNPs diet (1 mg/kg). Moreover, Rathore et al. (2021) found that the inclusion of SeNPs (1 mg/kg) in Nile tilapia fish challenged with *A. hydrophila* considerably enhanced the values of growth traits, WG, SGR, ADG, and PER and reduced FCR.

Several parameters in fish media may induce oxidative stress, specifically low water quality, overcrowding, heavy metals, pathogenic microorganisms, and malnutrition (Dawood et al., 2020). ROS production causes lipid peroxidation by attacking the lipid in the immune cells, ending with the impairment of the cell DNA and, thereby, the tissue’s death (Zhang et al., 2020; Gupta and Verma, 2022).

SeNPs were reported to keep blood parameters at normal levels in Nile tilapia challenged by *S. iniae* infection, making the fish healthier and more robust (Neamat-Allah et al., 2019). In addition, the inclusion of 1 mg/kg SeNPs for a 28-day diet reversed the decreases in RBC, WBC, Hb, Ht, MCV, MCH, and MCHC in Caspian roach exposed to 0.5 mg/L malathion (Zahmatkesh et al., 2020).

Several studies reported significant decreases in serum AST, ALT, and ALP levels in Nile tilapia, common carp, Caspian roach, and grass carp fed SeNPs (Yu et al., 2020; Zahmatkesh et al., 2020). The obtained results confirmed the protective action of Bio-SeNPs on liver performance and other tissue organs in fish.

The antioxidant potential of Se released from SeNPs may be linked to the synthesis of selenoproteins, which are essential in building and for the activity of GPx (Rotruck et al., 1973; Kumar et al., 2020). Further, selenoproteins are connected to a good immunity status of catfish, enabling resistance against various stressors.

In the current study, Bio-SeNPs have been revealed to have a special role in improving intestinal health and achieving maximum feed utilization, reflecting growth performance. Furthermore, the antibacterial potential of Bio-SeNPs may reduce pathogens present in fish intestines, increasing the efficiency of beneficial bacteria to digest the nutrients favoring good feeding behavior and avoiding bad ones. The promoting action of Bio-SeNPs on fish growth performance and feeding behavior can be partially attributed to the activation effect of Se on the digestive enzymes and improving the efficiency of intestinal villi (Dawood et al., 2021). Intestinal morphometry, villi length, and goblet cell number in Nile tilapia fish were reported to be enhanced by SeNPs (Ghazi et al., 2022).

Selenoproteins and GPx transport Se to the testis, forming an essential role in spermatogenesis (Imai et al., 2009). Additionally, Se has a role in male reproduction, incorporated in the mitochondrial capsule protein, developing spermatozoa motility and function (Boitani and Puglisi, 2008; Moslemi and Tavanbakhsh, 2011). Therefore, it is a potential solution for male sterility (Shi et al., 2010). Several studies have reported that the inclusion of SeNPs (0.3 mg/kg body weight) significantly improved Se content in the testis, the activity of semen GPx, and ATPase in male boar goats. Additionally, Shi et al. (2010) ensured the effect of SeNPs in protecting the membrane efficiency and the tight arrangement of the midpiece of the mitochondria. Hence, SeNPs appear more effective in enhancing male reproduction than the single Se element.

Hozyen et al. (2020) observed the adverse effects of deltamethrin (DLM) in male rats on sperm characteristics, testosterone, and antioxidant biomarkers, as well as behavioral and histopathological alterations. The Bio-SeNPs-treated groups improved semen parameters, antioxidant status, and sexual performance. Based on these observations, it can be concluded that Bio-SeNPs in aquafeed can enhance feeding and sexual behaviors, leading to increased fish yield. Therefore, SeNPs may represent an effective treatment for reducing the detrimental effects on male fertility, leading to enhanced reproductive performance.

## Conclusion

The accumulation of heavy metals and pathogenic microbes in fish organs is a severe problem, adversely affecting growth performance and productivity. The accumulation of heavy metals in fish organs causes oxidative stress by producing ROS, which damages cells and causes a low survival rate. Additionally, pathogenic bacteria accumulate in the gut and interfere with beneficial microorganisms, causing malnutrition, which affects growth performance. In this study, Bio-SeNPs synthesized by *Bacillus subtilis* AS12 isolate were proven with maximum efficiency as a remedy for heavy metal treatment and pathogen accumulation in fish organs and media. The unique characteristics of the obtained Bio-SeNPs, especially the negative charge and bioactive compounds’ coat, provide their antioxidant and antibacterial activity. Fish pathogens and heavy metal accumulation in fish media can be mitigated by including Bio-SeNPs (3–5 μg ml^−1^) in fish media, where they can improve the growth performance and survival rate of fish grown in regular or heavy metal-stressed media. Also, it maintained liver function at normal levels, enhancing the innate immune responses, GH, male hormones, and XX hormone levels. It is recommended to use Bio-SeNPs (3–5 μg ml^−1^) in polluted water as an efficient remedy to mitigate the accumulation of heavy metals and pathogens in fish organs.

## Data availability statement

The raw data supporting the conclusions of this article will be made available by the authors, without undue reservation.

## Ethics statement

The animal study has been reviewed and approved by ZU-IACUC committee. was performed in accordance with the guidelines of the Egyptian Research Ethics Committee and the guidelines specified in the Guide for the Care and Use of Laboratory Animals (2022). Ethical code number ZU-IACUC/2/F/394/2022. Written informed consent was obtained from the owners for the participation of their animals in this study.

## Author contributions

AS, MS, MS-A, KE-T, and ME-S, conceived and designed the experiments, analyzed the data, drafted the manuscript, and wrote and edited the final manuscript. All authors contributed to the article and approved the submitted version.

## Conflict of interest

The authors declare that the research was conducted in the absence of any commercial or financial relationships that could be construed as a potential conflict of interest.

## Publisher’s note

All claims expressed in this article are solely those of the authors and do not necessarily represent those of their affiliated organizations, or those of the publisher, the editors and the reviewers. Any product that may be evaluated in this article, or claim that may be made by its manufacturer, is not guaranteed or endorsed by the publisher.

## Footnotes

^1^ https://www.ncbi.nlm.nih.gov
