# Supplementary material for: Green nanotechnology for controlling bacterial load and heavy metal accumulation in Nile tilapia fish using biological selenium nanoparticles biosynthesized by Bacillus subtilis AS12
Source: Front Microbiol. 2022 Dec 23;13:1015613. doi: 10.3389/fmicb.2022.1015613 (PMC9816870; doi:10.3389/fmicb.2022.1015613)
Supplement: Supplementary file 1 [file Data_Sheet_1.pdf]

Supplementary material

Green nanotechnology for controlling bacterial load and heavy metal accumulation in Nile tilapia fish using biological selenium nanoparticles biosynthesized by *Bacillus subtilis* AS12

Ahmed M. Saad, Mahmoud Z. Sitohy, Mohamad I. Sultan-Alolama, Khaled A. El-Tarabily* and Mohamed T. El-Saadony.

*** Correspondence:**

Khaled A. El-Tarabily
ktarabily@uaeu.ac.ae

Supplementary material

Figure S1. Neighbor-Joining phylogeny of the *Bacillus subtilis* species. Numbers at branch points indicated bootstrap percentages. Only species that have percentage identities greater than 99.25% are listed.

Figure S2. Characterization of bio-selenium nanoparticles (Bio-SeNPs) (A) binding energy X-ray photoelectron spectroscopy (XPS), (B) X-ray diffraction (XRD) image, (C) Scanning electron microscopy (SEM) image.

Figure S3. Fourier transform-infrared (FTIR) spectroscopy spectra of (A) active groups in bio-selenium nanoparticles (Bio-SeNPs), (B) active groups in *Bacillus subtills* pellets supernatant.

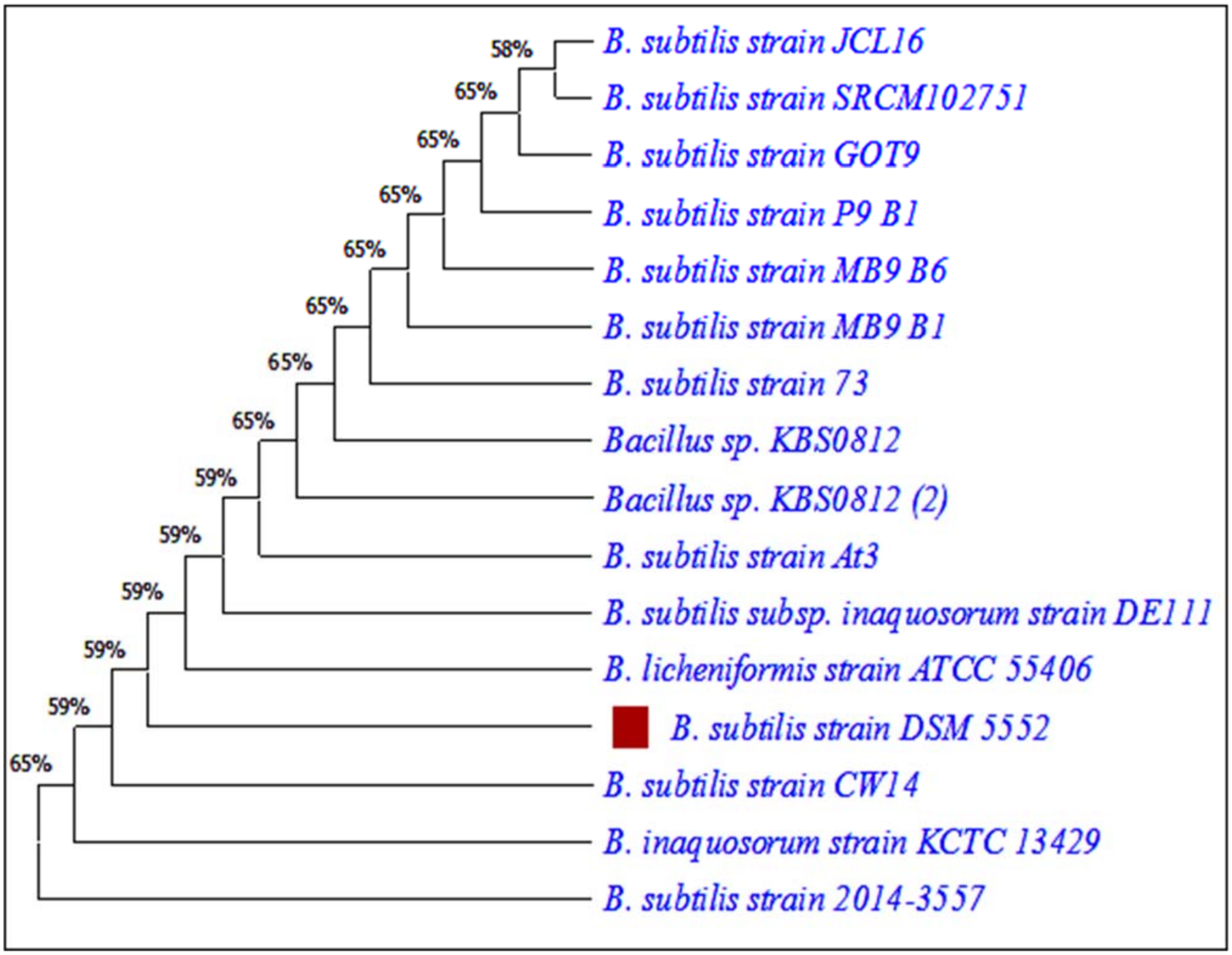

Figure S1. Neighbor-Joining phylogeny of the *Bacillus subtilis* species. Numbers at branch points indicated bootstrap percentages. Only species that have percentage identities greater than 99.25% are listed.

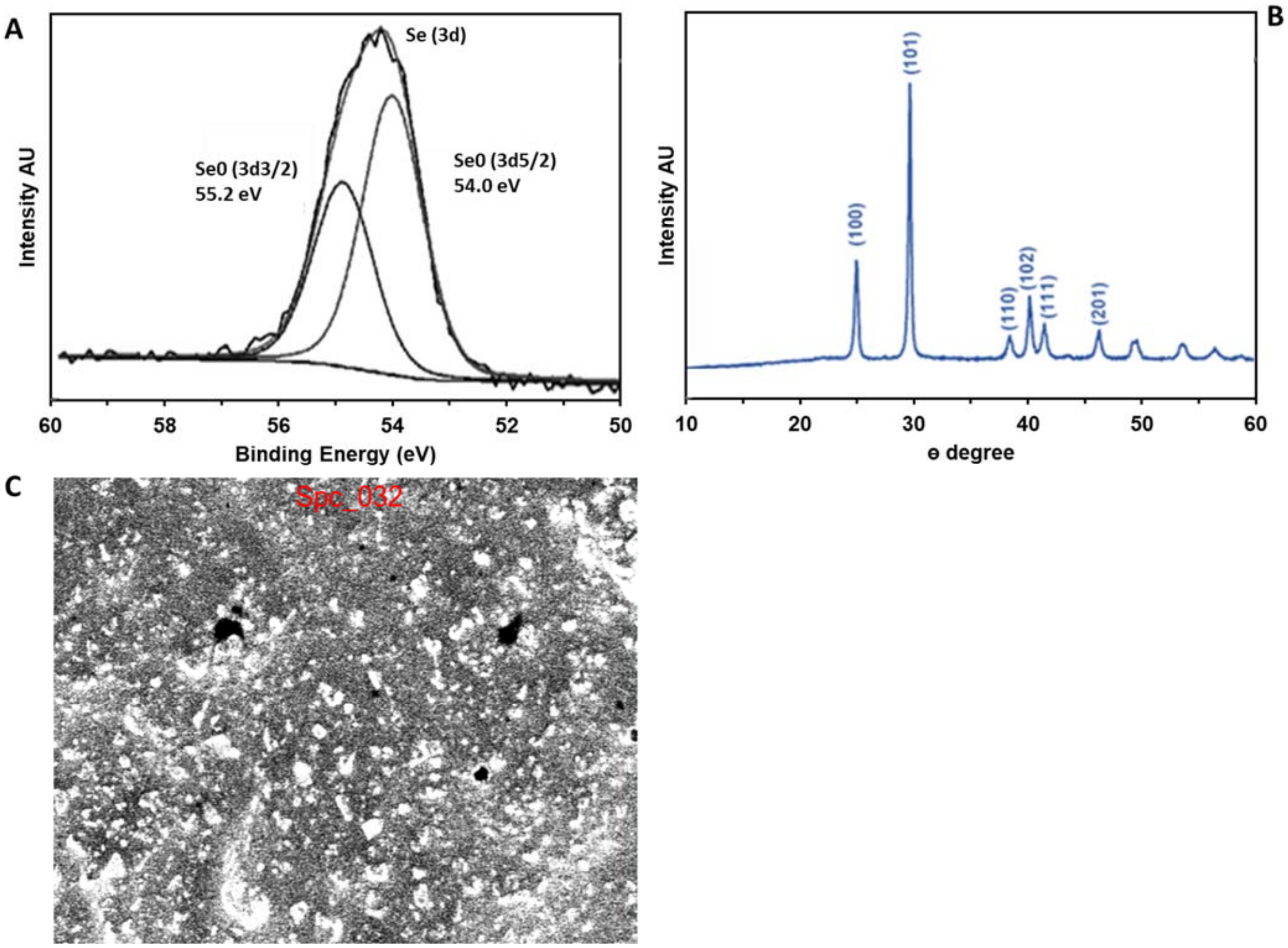

Figure S2. Characterization of bio-selenium nanoparticles (Bio-SeNPs) (A) binding energy X-ray photoelectron spectroscopy (XPS), (B) X-ray diffraction (XRD) image, (C) Scanning electron microscopy (SEM) image.

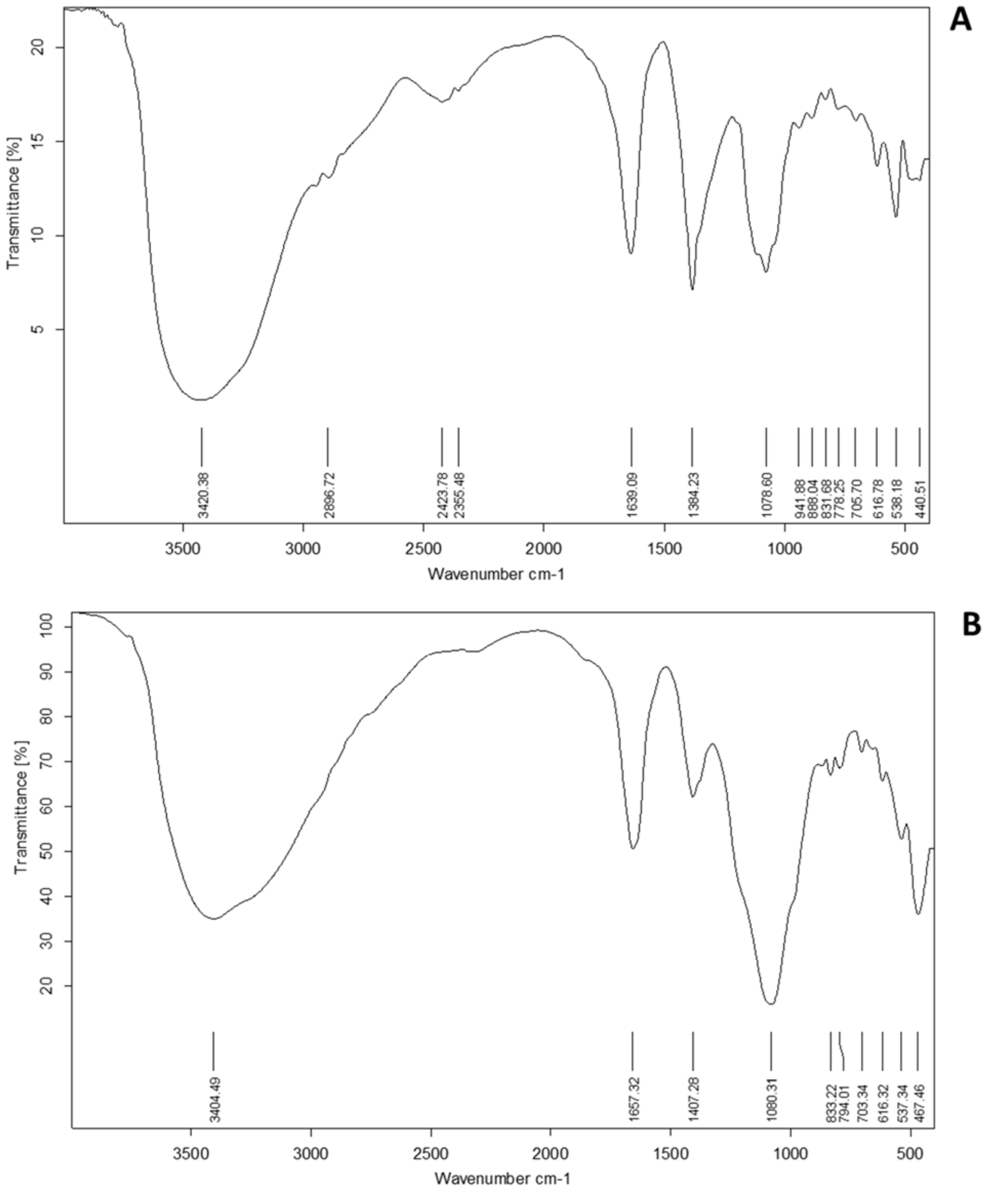

Figure S3. Fourier transform-infrared (FTIR) spectroscopy spectra of (A) active groups in bio-selenium nanoparticles (Bio-SeNPs), (B) active groups in *Bacillus subtills* pellets supernatant.
